# One‐step generation of heritable mitochondrial DNA multiplex‐engineered rats using DddA‐derived cytosine base editor

**DOI:** 10.1002/ame2.70154

**Published:** 2026-03-27

**Authors:** Xu Zhang, Keru Li, Lei Tan, Wei Chen, Shan Gao, Chenyang Liu, Shuo Pan, Jiayue He, Ning Liu, Gefan Wan, Wei Dong, Weining Kong, Bin Shen, Xiaolong Qi, Yuanwu Ma

**Affiliations:** ^1^ State Key Laboratory of Respiratory Health and Multimorbidity, NHC Key Laboratory of Human Disease Comparative Medicine, Key Laboratory of Pathogen Infection Prevention and Control Ministry of Education, Institute of Laboratory Animal Science Chinese Academy of Medical Sciences & Peking Union Medical College Beijing China; ^2^ State Key Laboratory of Reproductive Medicine Nanjing Medical University Nanjing Jiangsu China; ^3^ National Human Diseases Animal Model Resource Center and National Center of Technology Innovation for Animal Model, Haihe Laboratory of Cell Ecosystem, Institute of Laboratory Animal Science Chinese Academy of Medical Sciences & Peking Union Medical College Beijing China; ^4^ Medical Primate Research Center and Neuroscience Center Chinese Academy of Medical Sciences Beijing China

**Keywords:** DdCBE, mtDNA, mutation, rat

## Abstract

**Background:**

Mitochondrial DNA (mtDNA) mutations are implicated in a wide range of diseases, underscoring the need to elucidate the relationship between mtDNA mutations and disease pathology. These diseases are often characterized by the presence of multiple mutations; however, research has been hampered by the lack of suitable animal models carrying multiplex mtDNA mutations. Such models cannot be produced through traditional breeding due to matrilineal inheritance of mtDNA.

**Methods:**

Based on the TALE‐based mitochondrial genome editing tools, we generated rats harboring multiple mtDNA mutations by microinjecting mixed pairs of DdCBE plasmids into one‐cell‐stage zygotes. The efficiency of mtDNA editing and the potential off‐target effects were assessed through deep sequencing and long reads sequencing methodologies.

**Results:**

In this study, we established double‐ and triple‐site mutant rats with the editing efficiencies of up to 58.5% and confirmed that these mutations can be stably transmitted through the germline. Moreover, our results demonstrated that DdCBE‐mediated mtDNA multi‐site editing of mtDNA exhibits minimal off‐target effects in both the mitochondrial and nuclear genomes in vivo.

**Conclusion:**

This work represents the first successful generation of heritable multi‐site mtDNA mutant rats, providing a valuable model for elucidating the pathophysiological mechanisms of mitochondrial disorders and for developing potential therapeutics.

## Graphical abstract

We established that mixed DdCBE microinjection is an efficient, heritable, and precise strategy for generating multiplex mtDNA mutant rats. This advancement significantly expands the utility of DdCBEs for mitochondrial disease modeling, providing a robust platform for exploring the pathogenic mechanisms of complex mtDNA mutations and developing potential therapeutic interventions.

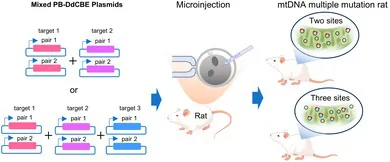

## 1 INTRODUCTION

Mitochondrial DNA (mtDNA) mutations play a crucial role in aging and in a variety of diseases.^1^, ^2^, ^3^, ^4^, ^5^, ^6^, ^7^ An increased accumulation of mtDNA mutations has been observed during aging process and is highly correlated with age.^8^ Although multi‐site mtDNA variations have been reported in various diseases and in aging,^9^, ^10^, ^11^ current research predominantly focuses on single‐site mtDNA mutations to establish genotype–phenotype correlations.^12^, ^13^, ^14^ Elucidating the synergistic interactions between multiple mtDNA mutations remains a significant yet underexplored challenge. The development of animal models carrying multi‐site mtDNA mutations would provide a powerful tool for dissecting the individual and combined effects on mitochondrial function, offering critical insights into disease mechanisms.

Recently, DdCBE (DddA‐derived cytosine base editor), which is engineered by fusing the toxin domain of the bacterial‐derived toxin deaminase (DddA) with the mitoTALE system, has been shown to efficiently induce C·G‐to‐T·A conversion in mtDNA in human cell lines,^15^, ^16^, ^17^, ^18^ zebrafish,^19^ mice,^14^, ^20^, ^21^ and rats.^22^, ^23^, ^24^ However, whether this system could be used to generate multiplex mtDNA‐engineered animal models requires further investigation. In this study, we extended the application of the DdCBE system to enable multiplex genetic engineering of the rat mitochondrial genome. We selected the m.7755G, m.14098G, m.007G, m.1030G, and m.11714/5G sites in rat mtDNA, which correspond to human pathogenic mutations (m.8363G>A, m.14710G>A, m.583G>A, m.1606G>A, and m.12315/6G>A), as targets for editing. In humans, point mutations in mitochondrial tRNA genes are associated with a spectrum of disorders. For instance, the m.8363G>A mutation in the *MT‐TK* gene is linked to myoclonus epilepsy with ragged‐red fibers (MERRF) syndrome, Leigh syndrome, ataxia, and lipomas.^25^, ^26^, ^27^, ^28^, ^29^, ^30^, ^31^ Other pathogenic variants include m.14710G>A in *MT‐TE*, associated with mitochondrial myopathy and retinopathy^32^; m.583G>A in MT‐TF, related to mitochondrial myopathy and asymptomatic retinopathy^33^; and m.12315/6G>A in *TRNL2*, which correlates with muscle degeneration syndrome and atherosclerosis.^34^, ^35^ Rats harboring two or three targeted mutations were successfully generated, achieving editing efficiencies of up to 58.5%. These multiplex‐edited rats demonstrated stable germline transmission of the mutations. Furthermore, off‐target analyses revealed that mixed DdCBE pairs enabled precise base editing with minimal off‐target effects in vivo. Our research substantially broadens the potential applications and enhances the versatility of mitochondrial base editing tools.

## 2 MATERIALS AND METHODS

### 2.1 Animals

The SD rats purchased from Beijing Vital River Laboratories Animal Technology Co., Ltd. were used in this study. All rats were maintained in the ILAS Animal facility with 12 h light/dark cycle and held under SPF conditions with free access to water and food. All animal procedures and experiments were approved by the Institutional Animal Care and Use Committee (IACUC) of the Institute of Laboratory Animal Science, Chinese Academy of Medical Sciences & Peking Union Medical College (IACUC‐MYW21006). All mutant rats established in this study were preserved at the Rat Resource Center of China (the rat resource website has changed from www.ratresource.com to https://ratresource.cnilas.org/) and the National Human Disease Animal Model Resource Center (https://namr.org.cn).

### 2.2 Microinjection of rat fertilized eggs

The fertilized rat oocytes were harvested and subjected to microinjection in accordance with the protocol previously outlined in reference.^22^, ^24^, ^36^ In summary, female rats aged 4–6 weeks were intraperitoneally administered with pregnant mare serum gonadotropin (PMSG, Sigma‐Aldrich) followed by human chorionic gonadotropin (hCG, Sigma‐Aldrich). After the hCG administration, the female rats were immediately paired for mating with male rats. The following morning, zygotes at the 1‐cell stage were retrieved from superovulated females and cultured in KSOM medium (Millipore) under conditions of 37°C and 5% CO_2_. The purified PB‐DdCBE pairs of plasmids constructed preciously^22^, ^24^ were microinjected into the cytoplasm of zygotes at a concentration of 8 ng/μL using a Nikon microinjection system, adhering to the manufacturer's protocol. Subsequently, the injected zygotes were transferred into pseudopregnant SD rats.

### 2.3 DNA extraction and genotyping

Genomic DNA from rat tissues was extracted using the EasyPure® Genomic DNA Kit (TransGen) following proteinase K digestion. The target regions were subsequently amplified and validated through Sanger sequencing and NGS. The primer sequences used in this study are detailed in Table S2.

### 2.4 Echocardiography analysis

The heart structure and function of rats were examined by echocardiography. In brief, the rat was anesthetized with isoflurane inhalation, and echocardiographic observation was performed using the Vevo3100 ultrasound machine with MX201 probe (15 MHz) (VisualSonics, Fujifilm). Measurements of at least three continuous cardiac cycles were recorded.

### 2.5 Open field test

Open Field Test was performed in 80 cm × 80 cm × 50 cm black box. Rat's spontaneous behavior was recorded for 5 min using a SuperMaze digital tracking system (version 2.1, Shanghai Xinruan Information Technology Co. Ltd, Shanghai, China). All tracks were analyzed by SuperMaze software.

### 2.6 Western blotting

Total protein of heart tissues was extracted with RIPA lysis buffer supplemented with protease inhibitor cocktail (P1006, Beyotime). Proteins were separated by 12% SDS‐PAGE following the standard procedures after quantified with a BCA protein assay kit (P0012S, Beyotime, China). Primary antibodies, such as anti‐UQCRC2 (ab14745, Abcam; 1:1000), anti‐COX2 (55070‐1‐AP, Proteintech; 1:1000), anti‐NDUFA1 (abcam ab131423; 1:1000), anti‐SDHA (ABclonal A2594; 1:1000), anti‐ATP5A (abcam ab176569; 1:1000), anti‐VDAC (Proteintech 66345‐1‐Ig; 1:2000), and anti‐GAPDH (HRP‐60004, Proteintech; 1:5000), were used. HRP‐conjugated secondary antibodies (CST) were diluted at a concentration of 1:5000. Protein signals were measured using an enhanced ECL Western blot Substrate (P0018S, Beyotime, China) and visualized with a Bio‐Rad imaging system.

### 2.7 ATP level and complex activity measurement

ATP levels in heart tissues were measured using an ATP Colorimetric Assay Kit (K354–100, BioVision, Inc.) as previously described.^24^ The complex activity of heart tissues was measured by the Mitochondrial Complex I/NADH‐CoQ reductase Activity Assay Kit (Solarbio, BC0515) and Mitochondrial Complex IV/Cytochrome C Oxidase Activity Assay Kit (Solarbio, BC0945) according to the kit instruction.

### 2.8 Deep‐sequencing

The target region was amplified using barcoded primers in a first round of polymerase chain reaction (PCR1), using Phanta Max Super‐Fidelity DNA Polymerase (P505, Vazyme). Subsequently, the PCR1 products were pooled in equimolar concentrations and subjected to purification in preparation for a second round of PCR (PCR2). The PCR2 process was conducted with index primers (N321/N322, Vazyme), and the products were purified using DNA Clean Beads (N411, Vazyme). Sequencing was carried out on the Illumina NovaSeq platform with the mean coverage of 8000× (>2 × 10^4^ reads/sample), and the quality score cutoff was Q30. The specific barcoded primers used in PCR1 are detailed in Table S2.

### 2.9 Whole mtDNA sequencing

Two overlapping fragments, each approximately 8 kb in length, were amplified using PrimerSTAR GXL DNA Polymerase (R050, Takara) with the primers designed for long‐range PCR. The amplicons were validated by 1% agarose gel electrophoresis and subsequently purified through gel extraction. For NGS, equimolar amounts of the two fragments were combined and processed for library preparation using the TruePrepTM DNA Library Prep Kit V2 (TD501, Vazyme). The resulting libraries underwent purification via DNA Clean Beads using a 0.5/0.3× double size selection strategy. These libraries were then pooled and sequenced using the Illumina NovaSeq platform with the mean coverage of 3000×, and the quality score cutoff was Q30. For Nanopore sequencing, library preparation was done using the ligation sequencing kit (SQK‐NBD114.24, Oxford Nanopore Technologies). All samples were sequenced using two R10.4.1 flow cells per sample on Oxford Nanopore PremethION sequencer monitored by MiniKNOW (v22.05.5) with the coverage of 4000×, and the quality score cutoff was Q10. Base calling of nanopore reads was done using the official basecaller termed Guppy (v3.2.1 and v5.0.7). The specific primers used for the long‐range PCR amplification are detailed in Table S2.

### 2.10 Data analysis

To assess the target editing frequencies, C·G‐to‐T·A efficiencies in the NGS data were analyzed using CRISPResso2.^37^ Base editing efficiencies were calculated as: base substitution reads divided by total reads. Target site editing efficiencies were calculated as: percentage of (the reads of C·G‐to‐T·A edits) / (total reads).

To calculate the editing frequency, sequencing reads were trimmed using Trim Galore in paired‐end mode and aligned to the *Rattus norvegicus* mitochondrial genome (NC_001665) with bowtie2's default paired‐end settings.^38^ The alignments were converted to BAM format using Samtools,^39^ and Samtools mpileup was used to detect DdCBE‐mediated C‐to‐T or G‐to‐A conversions. Single nucleotide polymorphism (SNP) sites were excluded based on the following criteria: (1) SNPs from *R. norvegicus* were sourced from Ensemble database's variation VCF; and (2) SNPs with C·G‐to‐T·A changes exceeding 1% in untreated sample were removed.

To determine the mitochondrial genome‐wide average off‐target editing frequency, editing events within the spacing region were omitted. SNP sites were excluded before analysis based on the following criteria: (1) SNP sites for *R. norvegicus* were identified from variation VCF in Ensemble database; (2) sites exhibiting C·G‐to‐T·A variation exceeding 1% in untreated samples; (3) sites showing a C·G‐to‐T·A variation greater than 90% in any sample; and (4) sites located within the DdCBE spacing region. The mean frequency of off‐target editing was subsequently determined independently for each biological replicate using the following formula: the sum of the reads in which a specific C·G base pair was identified as a T·A base pair, across all nontarget C·G base pairs, divided by the total number of reads encompassing all nontarget C·G base pairs.

## 3 RESULTS

### 3.1 DdCBE‐mediated generation of double‐site mtDNA mutations in rats

**FIGURE 1:**
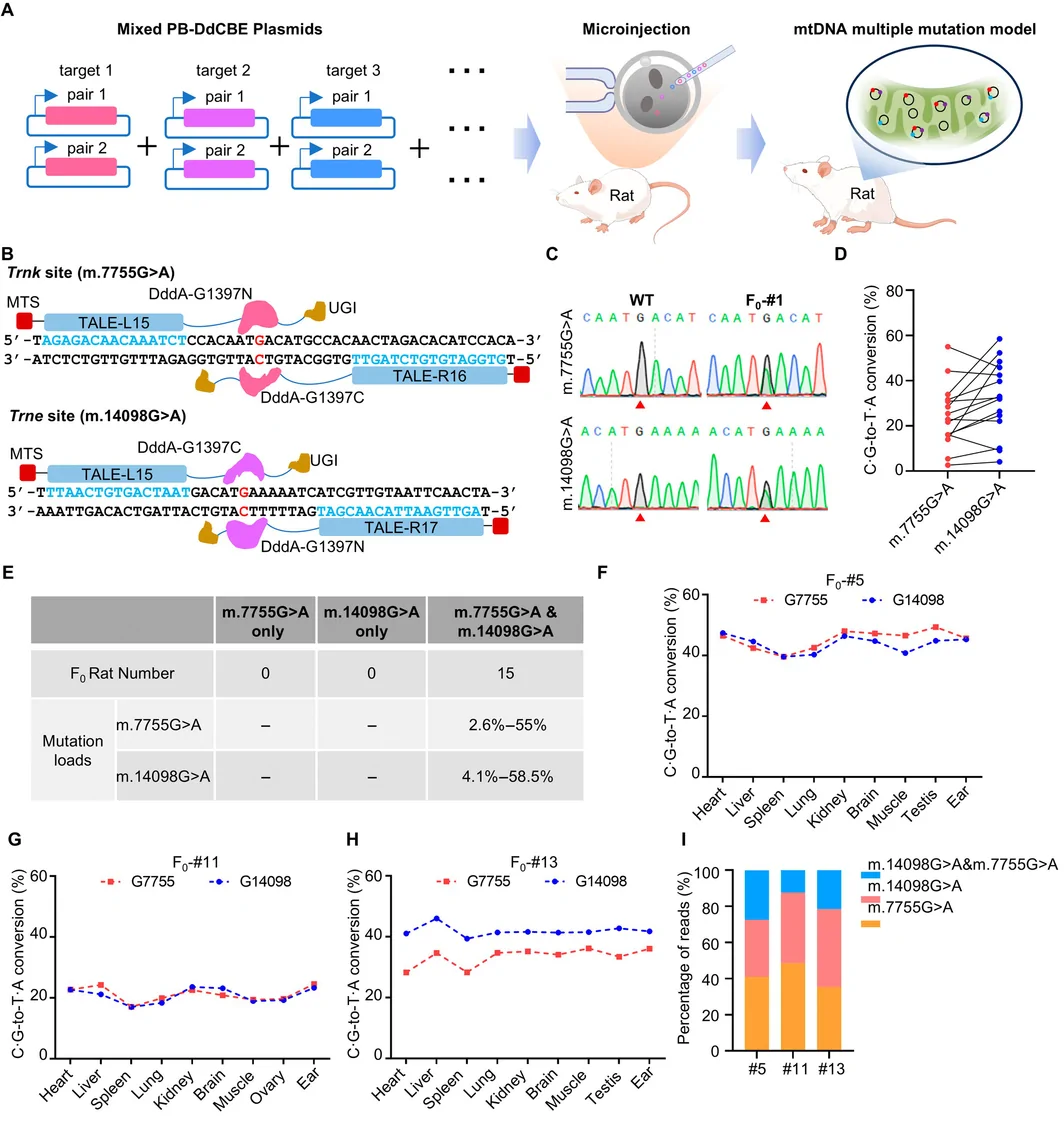
DdCBE‐mediated mtDNA double‐site editing in rats. (A) The strategy to generate mtDNA mutations at multiple sites in rats through microinjection of a mixture of DdCBEs. (B) The design of DdCBEs targeting to *TrnK* m.7755G and *TrnE* m.14098G sites. (C) The Sanger sequencing results of the *TrnK* m.7755G and *TrnE* m.14098G sites in F_0_‐#1 and WT rats. (D) Frequencies of G‐to‐A conversions at the *TrnK* m.7755G and *TrnE* m.14098G sites in F_0_ rats (*n* = 15). (E) Number of F_0_ rats that carried the *TrnK* m.7755G>A and *TrnE* m.14098G>A mutations. (F–H) The mutation loads in heart, liver, spleen, lung, kidney, brain, muscle, testis/ovary, and ear of F_0_‐#5 (F), #11 (G), and #13 (H) rats. (I) Allelic analysis results of *TrnK* m.7755G and *TrnE* m.14098G sites in double‐site mutant F_0_ (#5, #11, and #13) rats.

Mitochondrial DNA mutations exhibit considerable diversity and heterogeneity, which complicates investigations of mtDNA genotype and phenotype correlations.^40^, ^41^ To date, no animal models carrying multi‐site mtDNA mutations generated through precise mtDNA editing tools have been reported. To establish a rat model harboring multiple mtDNA mutations, we microinjected a mixture of validated DdCBE pairs of plasmids into fertilized rat zygotes to generate rats with multi‐site mtDNA mutations in one step (Figures 1A and S1). Initially, we microinjected a 1:1 mixture of PB‐DdCBE plasmids targeting the rat m.7755G site and the m.14098G site at a final concentration of 8 ng/μL for each plasmid (Figure 1B). A total of 15 founder (F_0_) rats were obtained (Table 1). Sanger sequencing confirmed that all 15 F_0_ rats harbored both the m.7755G>A and m.14098G>A mutations (Figures 1C and S2A). Next, we performed next‐generation sequencing (NGS) to determine the frequency of C·G‐to‐T·A conversion at each target site in the F_0_ rats. The results showed that the editing efficiency at the mtDNA m.7755G site ranged from 2.6% to 55%, whereas the efficiency at the m.14098G site ranged from 4.1% to 58.5% (Figure 1D,E).

**TABLE 1:** Injection summary of DdCBE‐mediated rat mtDNA editing.

Target site	Con. (ng/μL per target)	No. of injected zygotes	No. of transplanted zygotes	No. of pups	No. of edited pups
m.7755G/14098G	8	92	72	15	15
m.007G/1030G/11714/5G	8	175	112	23	23

To assess tissue distribution, we analyzed editing efficiencies in various tissues from three representative mutant F_0_ rats (F_0_‐#5, F_0_‐#11, and F_0_‐#13). NGS results indicated that the mutation rates for m.7755G>A or m.14098G>A were consistent across different tissues (Figures 1F–H and S2B). To evaluate potential editing bias at the two sites within individual mtDNA molecules, we conducted long‐read sequencing using Oxford Nanopore Technologies. The data revealed that single‐site editing events predominated: editing at the m.7755G alone accounted for 41.63% ± 6.63%, editing at the m.14098G site alone for 37.90% ± 5.88%, and simultaneous editing at both sites for 20.46% ± 7.61% (Figure 1I). Together, these results demonstrate that DdCBE‐mediated multiplex base editing can successfully generate rats carrying double‐site mtDNA mutations through one‐step microinjection.

### 3.2 DdCBE‐mediated generation of triple‐site mtDNA mutation in rats

**FIGURE 2:**
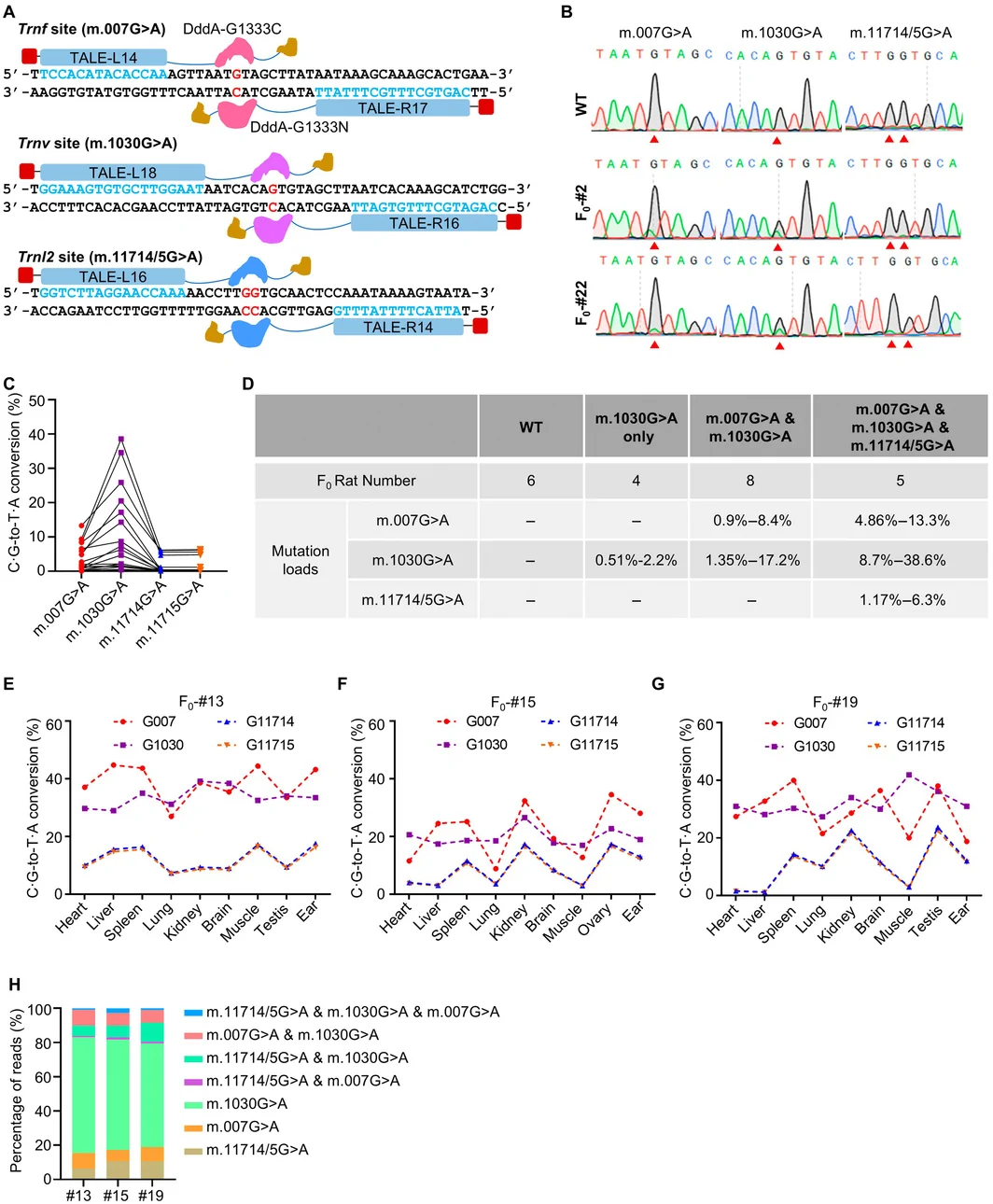
DdCBE‐mediated mtDNA triple‐site editing in rats. (A) The design of DdCBEs targeting to *TrnF* m.007G, *TrnV* m.1030G, and *TrnL2* m.11714/5G sites. (B) The Sanger sequencing results of the *TrnF* m.007G, *TrnV* m.1030G, and *Trn L2* m.11714/5G sites in F_0_ (#2 and #22) and WT rats. (C) Frequencies of G‐to‐A conversions at the *TrnF* m.007G, *TrnV* m.1030G, and *Trn L2* m.11714/5G sites in F_0_ rats (*n* = 17). (D) Number of edited F_0_ rats that carried the *TrnF* m.007G>A, *TrnV* m.1030G>A, and *Trn L2* m.11714/5G>A mutations. (E–G) The mutation loads in the heart, liver, spleen, lung, kidney, brain, muscle, testis/ovary, and ear of F_0_‐#13 (E), #15 (F), and #19 (G) rats. (H) Allelic analysis results of *TrnF* m.007G, *TrnV* m.1030G, and *Trn L2* m.11714/5G sites in triple‐site mutant F_0_ (#13, #15, and #19) rats.

To further assess whether this approach can be extended to generate rat models harboring more than two mtDNA mutations, PB‐DdCBE plasmids targeting the m.007G, m.1030G, and m.11714/5 G sites were mixed at a ratio of 1:1:1 and microinjected into fertilized rat zygotes at a final concentration of 8 ng/μL for each plasmid (Figure 2A). A total of 23 F_0_ rats were obtained following embryo transplantation (Table 1). Sanger sequencing confirmed that these F_0_ rats harbored the three target mutations with varying mutation rates (Figures 2B and S3A). The efficiency of C·T‐to‐G·A conversion at each target site was subsequently analyzed by NGS. The NGS results revealed that five F_0_ rats carried the m.007G>A, m.1030G>A, and m.11714/5G>A triple mutations. Among these, the mutation rates for m.007G>A ranged from 4.86% to 13.3%, for m.1030G>A from 8.7% to 38.6%, and for m.11714/5G>A from 1.17% to 6.13%. In addition, eight rats carried mutations at both the m.007G site (0.9%–8.4%) and the m.1030G site (1.35%–17.2%), and four rats harbored only the m.1030G>A single‐site mutation, with an editing efficiencies range from 0.51% to 2.2% (Figure 2C,D). To evaluate tissue‐specific distribution, we examined the editing efficiencies in different tissues of three representative F_0_ rats (F_0_‐#13, F_0_‐#15, and F_0_‐#19). The NGS data showed that the mutation rates at m.007G, m.1030G, and m.11714/5G sites were comparable across different tissues (Figures 2E–G and S3B). Furthermore, we performed long‐read mtDNA sequencing using the Oxford Nanopore Technologies to assess the editing bias in triple‐site edited rats. The results showed that the frequency of simultaneous editing at three sites within a single mtDNA molecular was 1.60% ± 1.03%. Editing at the m.007G site alone occurred at 8.08% ± 1.31%, at the m.1030G site alone at 64.07% ± 3.58%, and at the m.11714G site alone at 9.22% ± 2.45%, Dual‐site editing frequencies were 8.01% ± 1.09% for m.007G & m.1030G, 1.12% ± 0.32% for m.007G & m.11714/5G, and 7.90% ± 2.77% for m.1030G & m.11714/5G (Figure 2H). Collectively, these findings demonstrate that rat models with more than two mtDNA mutations can be generated through microinjection of mixed DdCBE system.

### 3.3 The multiplex mtDNA mutant rats were heritable

**FIGURE 3:**
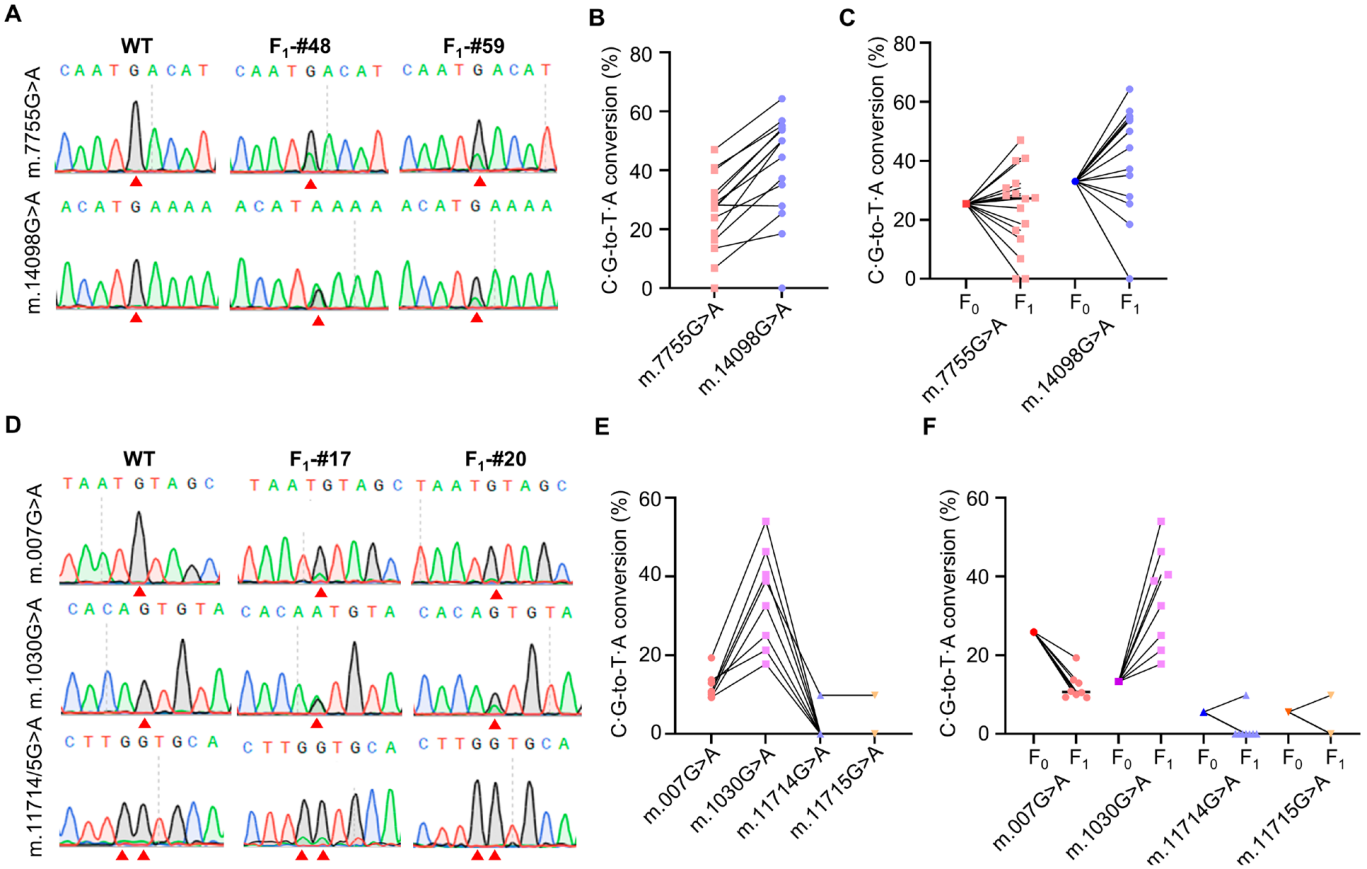
Germline transmission analysis of F_0_ mutant rats. (A) The Sanger sequencing results of the m.7755G and m.14098G sites in double‐site mutant F_1_ (#48 and#59) and WT rats. (B) Frequencies of G‐to‐A conversions at the m.7755G and m.14098G sites in double‐site mutant F_1_ rats (*n* = 16). (C) The variation of m.7755G>A and m.14098G>A mutations from double‐site mutant F_0_ rat to F_1_ rats (*n* = 16). (D) The Sanger sequencing results of the m.007G, m.G1030G, and m.1714/5G sites in triple‐site mutant F_1_ (#17 and #20) and WT rats. (E) Frequencies of G‐to‐A conversions at the m.007G, m.G1030G, and m.1714/5G sites in triple‐site mutant F_1_ rats (*n* = 8). (F) The variation of m.007G>A, m.1030G>A, and m.11714/5G>A mutations from triple‐site mutant F_0_ rat to F_1_ rats (*n* = 8).

**FIGURE 4:**
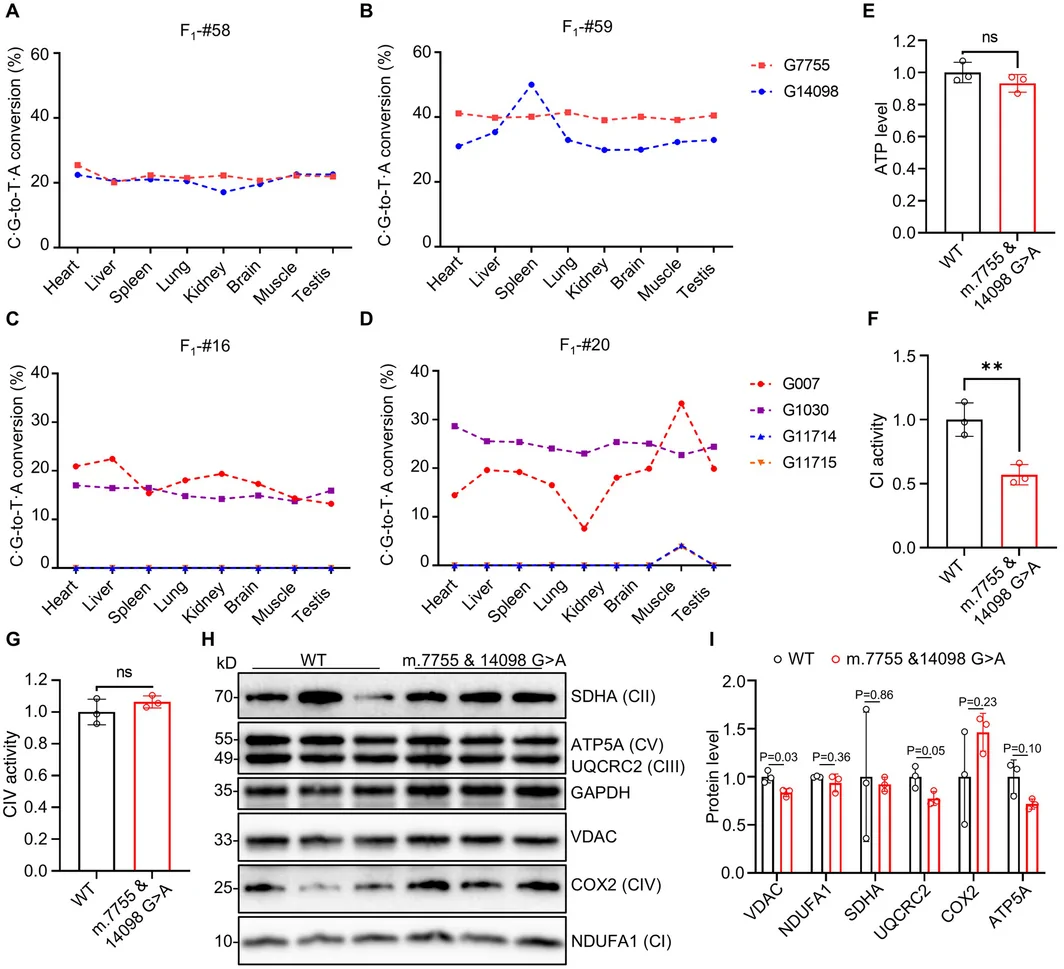
Analysis of mutation loads and mitochondrial function in F_1_ rats with mtDNA multiple mutations. (A and B) The mutation rates at the m.7755G and m.14098G sites in the heart, liver, spleen, lung, kidney, brain, muscle, and testis of double‐site mutant F_1_‐#58 (A) and F_1_‐#59 (B) rats. (C and D) The mutation rates at the m.007G & m.1030G & m.11714/5G sites in the heart, liver, spleen, lung, kidney, brain, muscle, and testis of triple‐site mutant F_1_‐#16 (C) and F_1_‐#20 (D) rats. (E) The ATP levels in heart tissues from WT and double‐site mutant F_1_ rats. *N* = 3 for each group; quantitative data were analyzed using the two‐tailed unpaired Student's *t*‐test and are presented as means ± SD. ns, nonsignificant. (F and G) The activity of CI (F) and CIV (G) in heart tissues from WT and double‐site mutant F_1_ rats. *N* = 3 for each group; quantitative data were analyzed using the two‐tailed unpaired Student's *t*‐test and are presented as means ± SD. ns, nonsignificant; **p < 0.01. (H and I) The western blot (H) and quantitative analysis (I) of five complex protein levels in heart tissues from WT and double‐site mutant F_1_ rats. *N* = 3 for each group. Quantitative data were analyzed with the two‐tailed unpaired Student's *t*‐test and are presented as means ± SD.

Given that mtDNA is maternally inherited, we selected a double‐mutation female rat (F_0_‐#10) and a triple‐mutation female rat (F_0_‐#22) to assess whether the introduced mutations could be transmitted to the next generation. These females were mated with wild‐type Sprague–Dawley (SD) males. As a result, 16 F_1_ rats carrying double mutations and 8 F_1_ rats carrying triple mutations were obtained. Sanger sequencing confirmed that all 16 F_1_ rats inherited the m.7755G>A & m.14098G>A double mutations (Figures 3A and S4), with mutation rates from 6.85% to 47.106% for m.7755G>A and from 18.52% to 64.29% for m.14098G>A, respectively (Figure 3B,C and Table S1). Among the eight F_1_ rats derived from the triple‐mutation founder, one individual (#17) carried all three target mutations—m.007G>A, m.1030G>A, and m.11714/5G>A—with mutation rates of 10%, 38.89%, and 9.8%, respectively (Figures 3D and S5). The remaining seven F_1_ rats inherited only two of the three mutations, with m.007G>A mutation rates ranging from 9.21% to 19.32% and m.1030G>A rates ranging from 17.78% to 54.05% (Figure 3E,F and Table S1). To further evaluate tissue‐specific transmission, we analyzed the mutation rates in various tissues from two representative F_1_ rats. Both Sanger sequencing and NGS analyses revealed that the mutation rates were relatively consistent across different tissues within each individual, despite some inter‐individual variation among the progeny carrying double and triple mutations (Figures 4A–D and S6A). Collectively, these results confirm that the multiplex mtDNA mutations generated by DdCBE‐mediated editing can be stably inherited through the maternal lineage.

In our previous work, we demonstrated that the m.14098G>A mutation impairs cardiac and skeletal muscle function in rats.^22^ Here, we further investigated the combined effects of the m.7755G>A & m.14098G>A double mutations on cardiac and muscle phenotypes in F_1_ rats at 8 weeks of age. Echocardiographic assessment showed no significant differences in cardiac structure and function between double‐mutation F_1_ males and their wild‐type littermates (Figure S5C–J). In addition, the Open Filed Test indicated no significant difference in total locomotor activity between the two groups (Figure S6K), and grip strength measurements were also comparable (Figure S6L). We further analyzed ATP content and the enzymatic activities of Complex I (CI) and Complex IV (CIV) in heart tissues. Notably, CI activity was significantly reduced in the hearts of F_1_ rats with double mutations compared to WT rats (Figure 4E–G). However, the protein level of representative subunits NDUFA1 (CI), SDHA (CII), UQCRC2 (CIII), COX2 (CIV), ATP5A (CV), and the mitochondrial marker VDAC were comparable between double‐mutation F_1_ rats and WT controls (Figure 4H,I).

### 3.4 Off‐target effects of DdCBE‐mediated multiplex mtDNA editing

**FIGURE 5:**
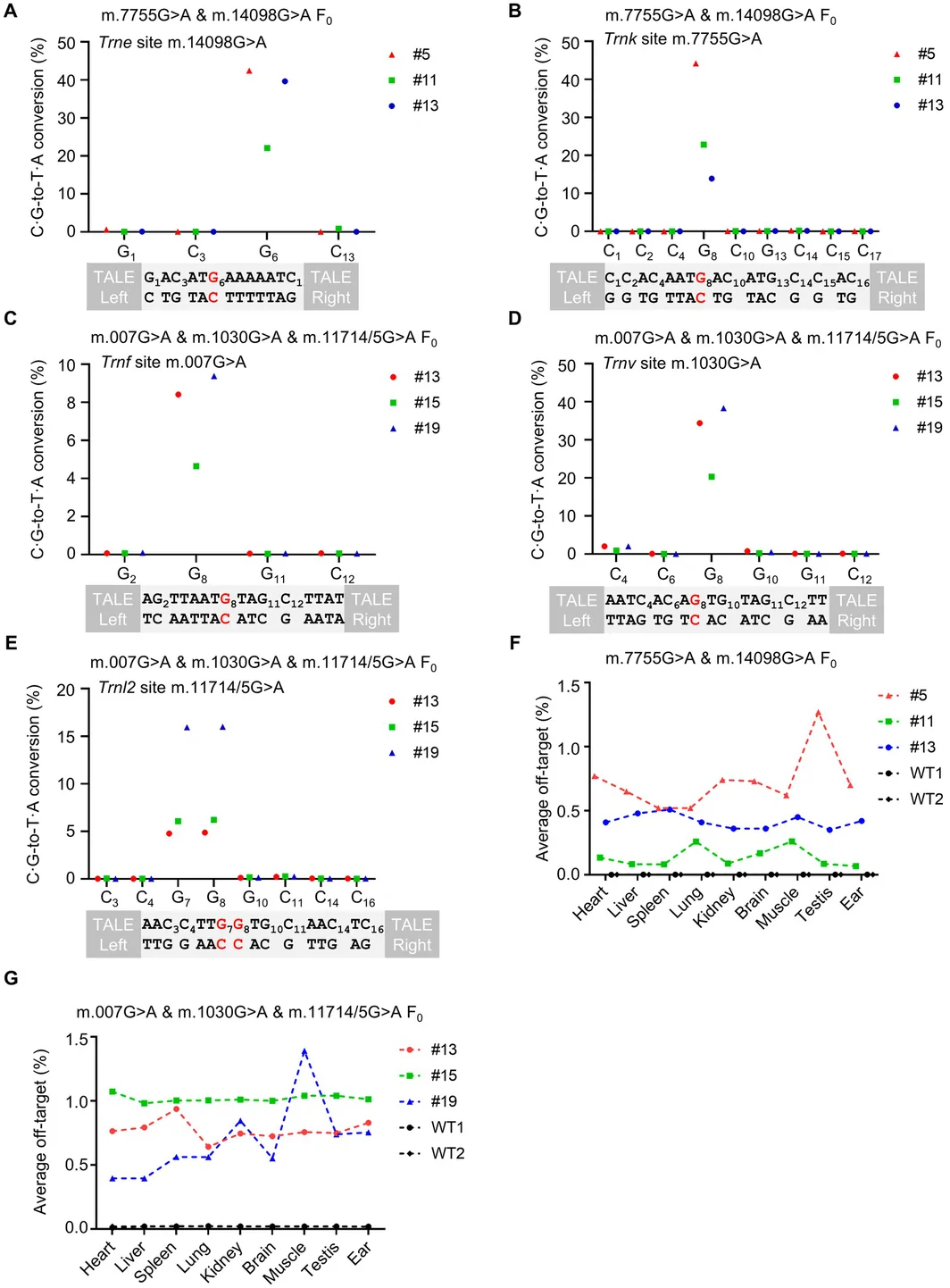
Off‐target analysis on the mitochondrial genome. (A and B) The frequencies of C· G‐to‐T ·A conversions in editing window of DdCBEs targeting m.14098G site (A) and m.7755G site (B) in double‐site mutant F_0_ rats (*n* = 3). (C–E) The frequency of C· G‐to‐T ·A conversion in editing window of DdCBEs targeting m.007G site (C), m.1030G site (D), and m.11714/5G site (E) in triple‐site mutant F_0_ rats (*n* = 3). (F) Average frequency of mitochondrial genome‐wide C· G‐to‐T ·A conversions in double‐site mutant F_0_ and WT rats. (G) Average frequency of mitochondrial genome‐wide C· G‐to‐T ·A conversions in three‐site mutant F_0_ and WT rats.

Potential off‐target effects represent a critical concern for any gene‐editing technology. In our previous studies, we demonstrated that the selected DdCBE pairs induce minimal off‐target modifications in rats.^22^, ^24^ To further assess the fidelity of multiplex mtDNA editing in this study, we first evaluated possible bystander edits within the spacer regions adjacent to the target sites (m.7755G, m.14098G, m.007G, m.1030G, and m.111714/5G) in both double‐ and triple‐mutation F_0_ rats. NGS revealed no detectable bystander editing events in three double‐mutation F_0_ rats (#5, #11, and #13) (Figure 5A,B) as well as in three triple‐mutation F_0_ rats (#13, #15, and #19) (Figure 5C–E). Subsequently, we conducted comprehensive whole‐mitochondrial genome sequencing on multiple tissues to evaluate global off‐target effects (Table 2). The results indicated that the average off‐target rate across the entire mtDNA was consistently below 1.5% in both double‐mutation F_0_ rats (#5, #11, and #13) (Figures 5F and S7A) and triple‐mutation F_0_ rats (#13, #15, and #19) (Figures 5G and S8A). Consistent with F_0_ rats, the mutation‐bearing F_1_ rats also exhibited extremely low mtDNA off‐target editing, with rates not exceeding 0.35% across multiple tissues for both double‐ and triple‐mutation progeny (Figures S7B and S8B).

**TABLE 2:** Genome‐wide mtDNA off‐target summary.

	F_0_		F_1_	
	m.7755G/14098G	m.007G/1030G/11714/5G	m.7755G/14098G	m.007G/1030G/11714/5G
Heart	0.44% (Means)	0.13%	0.74% (Means)	1.25%
	0.77% (Max)	0.20%	1.07% (Max)	1.79%
Liver	0.40%	0.07%	0.72%	0.66%
	0.65%	0.12%	0.98%	0.67%
Spleen	0.37%	0.08%	0.83%	1.64%
	0.52%	0.12%	1.00%	2.64%
Lung	0.40%	0.12%	0.74%	1.27%
	0.52%	0.20%	1.00%	1.38%
Kidney	0.40%	0.14%	0.87%	1.41%
	0.74%	0.32%	1.01%	2.22%
Brian	0.42%	0.09%	0.76%	0.72%
	0.73%	0.15%	1.00%	0.74%
Muscle	0.44%	0.07%	1.06%	1.14%
	0.62%	0.11%	1.04%	1.27%
Testicle	0.57%	0.07%	0.84%	1.04%
	1.27%	0.12%	1.04%	1.32%
Ear	0.40%	–	0.87%	–
	0.70%		1.01%	

In addition to mitochondrial off‐target events, it has been reported that DdCBEs can induce TALE‐dependent nuclear off‐target editing in the nuclear genome.^42^ To analyze the nuclear off‐target events, we performed deep‐sequencing analysis on potential nuclear off‐target sites (OTSs) that share identical or highly similar sequences with the TALE‐binding motifs. Specifically, six candidate OTSs were identified for the m.7755G (OTS1–6; Figure S9A), three for m.14098G (OTS1–3; Figure S10A), five for m.007G (OTS1–5; Figure S11A), two for m.1030G (OTS1–2; Figure S12A), and six for m.11714/5G (OTS1–6; Figure S13A). Deep‐sequencing analysis of these nuclear sites revealed no detectable TALE‐dependent off‐target editing events in any of the tested animals (Figures S9B, S10B, S11B, S12B, and S13B).

Taken together, these findings demonstrate that multiplex mtDNA editing via mixed DdCBE microinjection induces minimal off‐target effects in both mitochondrial and nuclear genomes. Therefore, this approach represents a precise, efficient, and safe strategy for generating multiplex mtDNA mutant rat models.

## 4 DISCUSSION

Although multi‐site mtDNA variations are implicated in various diseases and aging, their study has been largely inaccessible due to technical limitations.^6^, ^9^, ^10^, ^11^ Consequently, current research predominantly focuses on single mutations to establish genotype–phenotype links.^12^, ^13^, ^22^, ^24^ Investigating the synergistic effects of multiple mutations remains a significant and underexplored challenge. To address this, the development of animal models with multi‐site mtDNA edits would provide a powerful tool for dissecting individual and combined effects on mitochondrial function and disease mechanisms.

To establish this methodology, we selected several known pathogenic human mitochondrial tRNA point mutations for initial model development. We successfully expanded the application of DdCBEs to generate multiplex mtDNA mutation rat models by targeting rat homologs of pathogenic human sites: m.7755G (human m.8363G), m.14098G (human m.14710G), m.007G (human m.583G), m.1030G (human m.1606G), and m.11714/5G (human m.12315/6G). Through co‐injection of a DdCBE mixture into rat zygotes, we successfully generated double and triple mtDNA mutations in a single generation. In double‐mutant F_0_ rats, editing efficiencies reached up to 55% for m.7755G>A and 58.5% for m.14098G>A, which are notably higher than the efficiencies observed for single‐site edits (36.66% and 46.73%, respectively). In triple mutant F_0_ rats, the highest observed efficiencies were 13.3% for m.007G>A, 38.6% for m.1030G>A, and 6.13% for m.11714/5G>A. The efficiency at m.1030G>A also exceeded that observed in our previous single‐site editing experiments (24.1%), although editing efficiencies at m.007G>A and m.11714/5G>A were lower than those in their respective single‐site results (67.89% and 40.68%). Collectively, these findings demonstrate that multiplex mtDNA mutations can be efficiently generated via mixed DdCBE microinjection, despite site‐specific variations in editing efficiency.

Because mitochondria is strictly maternally inherited, traditional breeding cannot generate multiplex mtDNA mutant animals. Here, the multiple mutations introduced by mixed DdCBE injection were successfully transmitted to the next generation through maternal inheritance, enabling long‐term multigenerational studies. We also analyzed the distribution of multiple mutations in different tissues of F_1_ rats. The mutation loads in various tissues were relatively consistent for each targeted site. However, no significant alterations in cardiac or muscle function were observed. A recent study delineated that a general heteroplasmy threshold for pathogenicity is approximately 75%.^43^ In clinical cases, the m.8363G>A mutation manifests disease phenotypes in muscle tissues and brain at heteroplasmy levels of 80–98%,^30^, ^31^, ^44^, ^45^ whereas the m.14710G>A mutation shows pathology at levels of 92%–96%.^32^ Our data align with this principle. Despite a significant reduction in Complex I activity in the double‐mutant (m.7755G>A & m.14098G>A) F_1_ rats, they still had not yet reached the heteroplasmy threshold necessary to elicit observable phenotypic consequences.

Long‐read sequencing provides a detailed view of multiplex editing outcomes, confirming precise C‐to‐T conversion at individual target sites. However, the fraction of mtDNA molecules containing all intended simultaneous edits—termed co‐editing efficiency—is substantially lower than the efficiency observed for single sites. For distant loci, this likely reflects the combined kinetic and physical challenges of concurrent deaminase activity within the compact mitochondrial genome, coupled with the stochastic segregation of edited genomes. We propose several contributing factors: (1) in cis competition among DdCBE pairs for shared cellular resources or the mtDNA molecule itself, altering binding kinetics; (2) structural perturbations, such as changes in local supercoiling or protein occupancy induced by one editor, which may positively or negatively affect neighboring site accessibility—a form of structural epistasis; (3) divergent kinetics arising from the co‐expression of multiple editors, including variations in RNA stability or deaminase activation; and (4) the stochastic bottleneck of mitochondrial segregation during early embryogenesis, which shapes the final heteroplasmy in founder animals. Together, these observations underscore that multiplex editing efficiency is not a simple aggregate of individual events but an emergent property of a complex system. Understanding these interactions offers a roadmap for optimization, such as fine‐tuning editor expression or selecting structurally synergistic target sites. Breeding strategies can further preserve the model's utility by enriching for animals with higher heteroplasmy of the combined mutations.

We also comprehensively assessed off‐target effects in both the mitochondrial and nuclear genomes. Whole mtDNA sequencing showed that the average off‐target frequency in multiplex F_0_ rats did not exceed 1.5%, which is comparable to or only slightly higher than the ~1% observed in our previous single‐mutant models. Moreover, this frequency decreased to below 0.35% in F_1_ progeny, indicating a dilution effect over generations. In addition, no detectable TALE‐dependent off‐target editing was found in the nuclear genome of multiplex mutant rats. These results demonstrate that co‐injecting multiple DdCBEs maintains high editing precision and genomic safety.

In summary, our study establishes that mixed DdCBE microinjection is an efficient, heritable, and precise strategy for generating multiplex mtDNA mutant rats. This advancement significantly expands the utility of DdCBEs for mitochondrial disease modeling, providing a robust platform for exploring the pathogenic mechanisms of complex mtDNA mutations and developing potential therapeutic interventions.

In this study, we successfully generated rats harboring double and triple mtDNA mutations using DdCBE‐mediated multiplex editing. However, several limitations remain to be addressed. First, the feasibility and efficiency of this approach need to be validated in other animal species to confirm its generalizability beyond rats. Second, the current method targets only *tC* and *aC* contexts, and the editing efficiency at each site depended on the transient expression and enzymatic activity of the injected DdCBE pairs in one‐cell embryos, which may vary between loci. Third, although we achieved stable germline transmission, we did not establish a clear link between the heteroplasmic mutations and overt pathological phenotypes. Future studies should aim to design combinations of mtDNA mutations that more closely mimic clinically relevant co‐existing mutations in humans and to employ improved delivery strategies—such as mRNA or circular RNA (circRNA)—to enhance editing efficiency and minimize mosaicism. The covalently closed structure of circRNA provides exceptional stability by protecting it from exonuclease degradation. This key attribute makes circRNA a compelling candidate for advanced RNA‐based therapeutics and delivery systems, most notably in SARS‐CoV‐2 vaccine development.^46^, ^47^ Furthermore, next‐generation high‐fidelity DddA variants—such as DddA6 and DddA11, evolved via phage‐assisted continuous evolution—demonstrate notably improved editing activity. Their use can significantly increase on‐target efficiency while reducing off‐target effects, making them versatile tools for advanced base editing.^48^ Advanced and more sensitive off‐target detection methods are also needed to comprehensively assess unintended edits in multiplex mtDNA mutant models. Finally, the development of novel mitochondrial editing tools with broader sequence compatibility, higher efficiency, and improved specificity remains a priority. Such advancements will help achieve higher mutation loads and more robust genotype–phenotype correlations, ultimately strengthening the utility of multiplex mtDNA mutant rats for modeling human mitochondrial diseases and testing therapeutic interventions.

## AUTHOR CONTRIBUTIONS

**Xu Zhang:** Data curation; investigation; validation. **Keru Li:** Investigation. **Lei Tan:** Formal analysis; investigation. **Wei Chen:** Investigation. **Shan Gao:** Investigation. **Chenyang Liu:** Investigation. **Shuo Pan:** Investigation. **Jiayue He:** Investigation. **Ning Liu:** Investigation. **Gefan Wan:** Formal analysis; investigation; validation. **Wei Dong:** Investigation. **Weining Kong:** Investigation. **Bin Shen:** Supervision. **Xiaolong Qi:** Conceptualization; investigation; methodology; visualization; writing – original draft; writing – review and editing. **Yuanwu Ma:** Conceptualization; methodology; project administration; resources; supervision.

## FUNDING INFORMATION

This work was supported by Chinese Academy of Medical Sciences Innovation Fund for Medical Sciences (2021‐I2M‐1‐024, 2022‐I2M‐1‐020, and 2023‐I2M‐2‐001); the Non‐profit Central Research Institute Fund of the Chinese Academy of Medical Sciences (2023‐PT180‐01); Haihe Laboratory of Cell Ecosystem Innovation Fund (HH24KYZX0007); Open Research Project in State Key Laboratory of Vascular Homeostasis and Remodeling (Peking University) (202411); State Key Laboratory Special Fund (2060204); the National Key R&D Program of China (2022YFF0710702).

## CONFLICT OF INTEREST STATEMENT

Yuanwu Ma is an editorial board member of Animal Models and Experimental Medicine (*AMEM*) and a corresponding author of this article. To minimize bias, he was excluded from all editorial decision making related to the acceptance of this article for publication.

## CODE AVAILABILITY

The data analysis code used in this paper is available from GitHub (https://github.com/ilya0520/njmushenlab) and supplementary materials.

## ETHICS STATEMENT

All animal procedures and experiments were approved by the Institutional Animal Care and Use Committee (IACUC) of the Institute of Laboratory Animal Science, Chinese Academy of Medical Sciences & Peking Union Medical College (IACUC‐MYW21006).

## MATERIALS AVAILABILITY

The mtDNA mutation rat lines used in this paper are available from Rat Resource Center of China (https://ratresource.cnilas.org/) to other scientific researchers upon request.

## Supporting information


Figure S1‐S13. Table S1 and S2.

## Data Availability

The high‐throughput sequencing data to NCBI Sequence Read Archive (SRA) database (BioProject ID: PRJNA1212772 and PRJNA1212798). Any additional information required to reanalyze the data reported in this paper is available from the lead contact upon request.
