# Supplementary material for: One‐step generation of heritable mitochondrial DNA multiplex‐engineered rats using DddA‐derived cytosine base editor
Source: Animal Model Exp Med. 2026 Mar 27:10.1002/ame2.70154. Online ahead of print. doi: 10.1002/ame2.70154 (PMC13393515; doi:10.1002/ame2.70154)
Supplement: Supplementary file 1 — Figure S1‐S13. Table S1 and S2. [file AME2-9999-0-s001.pdf]

Figures

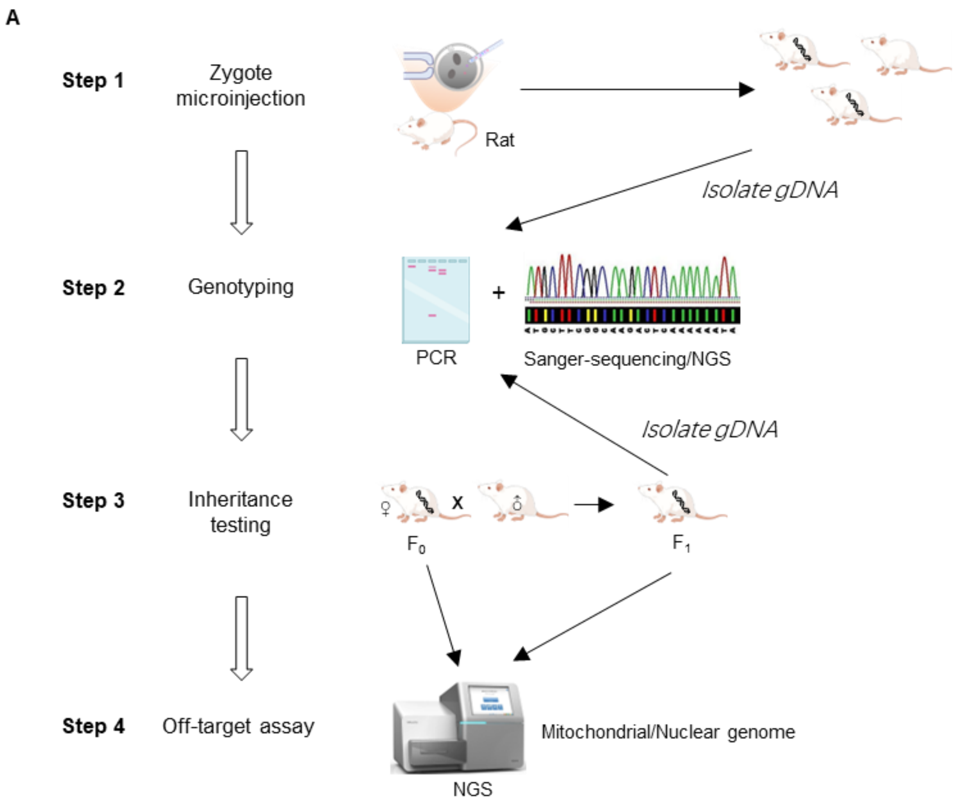

Figure S1. Schematic pipeline for the generation and validation of multi-site mtDNA mutant rat models.

(A) This flowchart summarizes the key experimental and analytical steps for creating and characterizing rat models with targeted mitochondrial DNA mutations. The pipeline begins with the microinjection of validated DdCBE pairs of plasmids into zygotes, followed by embryo transfer to produce founder (F_0) animals. Founder rats then undergo a multi-layered genotyping strategy, including initial PCR screening, deep sequencing to quantify target-site heteroplasmy, comprehensive nuclear off-target screening, and full mtDNA sequencing. Positive F_0 animals are outcrossed to wild-type rats to assess maternal inheritance patterns and heteroplasmy stability in the F_1 generation.

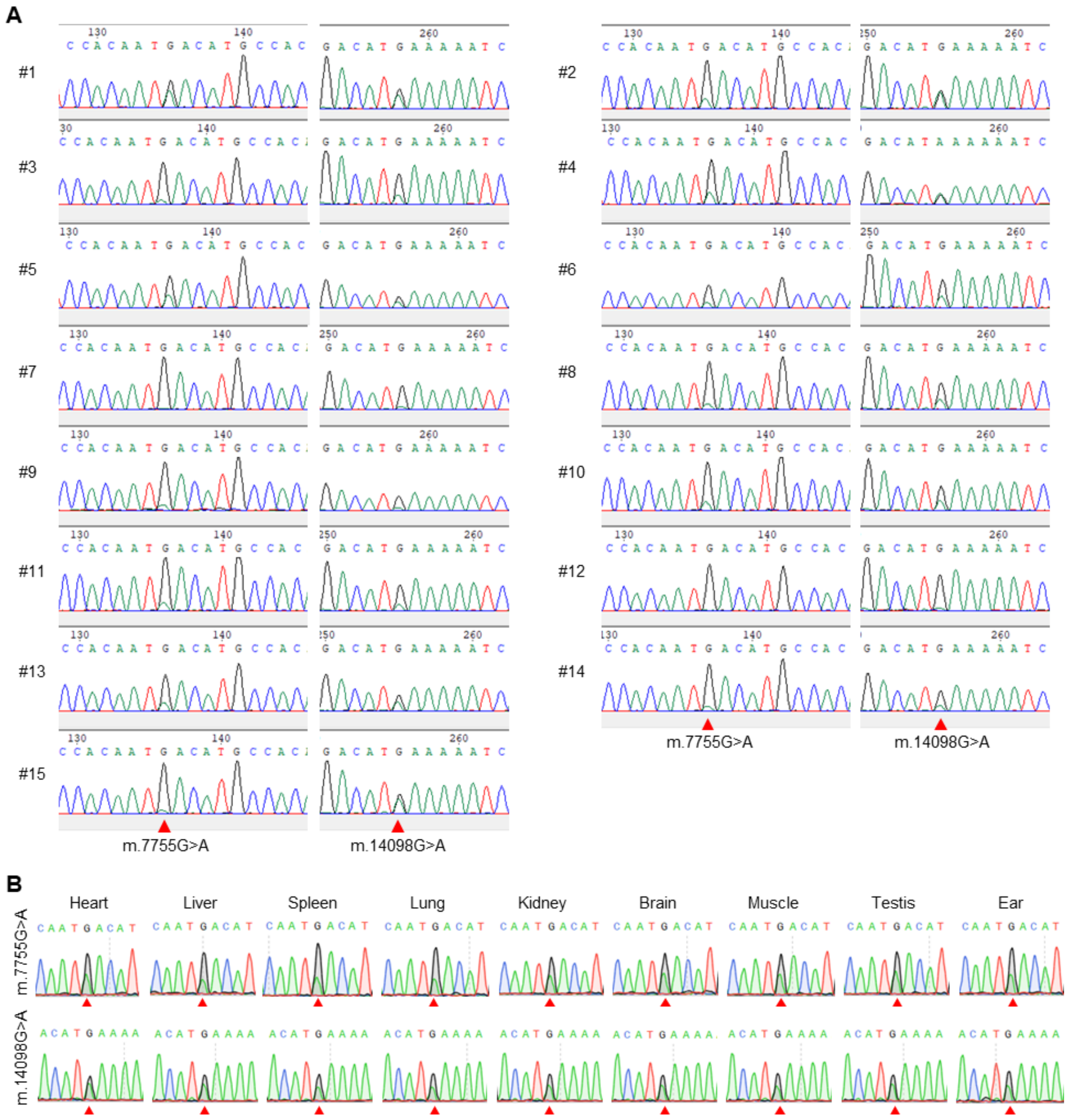

Figure S2. Mutation load in DdCBE-mediated mtDNA two-site editing in rats.

(A) Sanger sequencing chromatograms of the m.7755G and m. 14098G sites in the toes of fifteen F_0 rat with two-site editing.

(B) Sanger sequencing results of the m.7755G and m.14098G sites in heart, liver, spleen, lung, kidney, brain, muscle, testis, and ear of F_0-#5 rat.

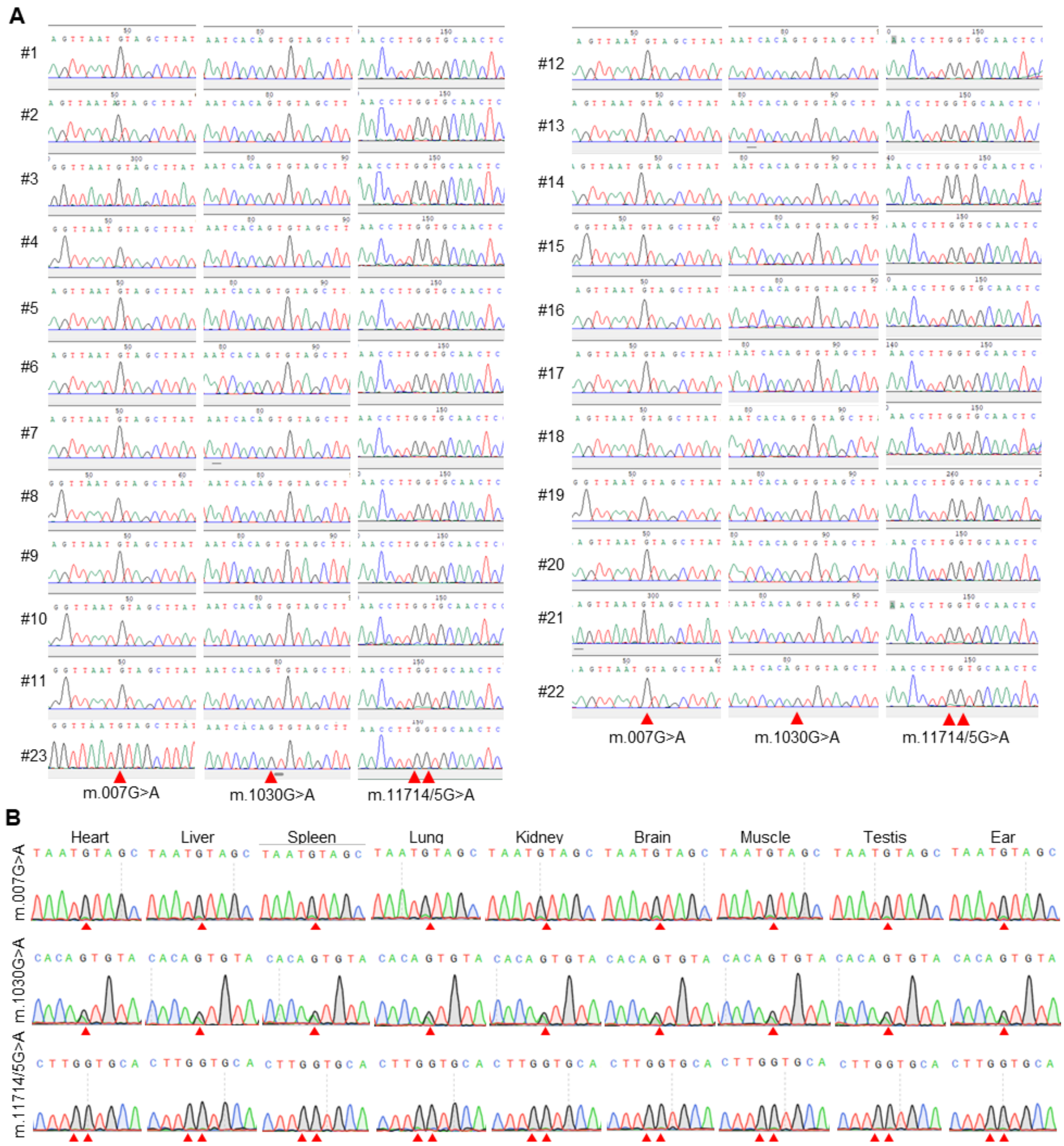

Figure S3. Mutation load in DdCBE-mediated mtDNA three-site editing in rats.

(A) Sanger sequencing chromatograms of the m.007G, m.1030G, and m.11714/5G sites in the toes of twenty-three F_0 rat with three-site editing.

(B) Sanger sequencing results of the m.007G, m.1030G, and m.11714/5G sites in heart, liver, spleen, lung, kidney, brain, muscle, testis, and ear of F_0-#13 rat.

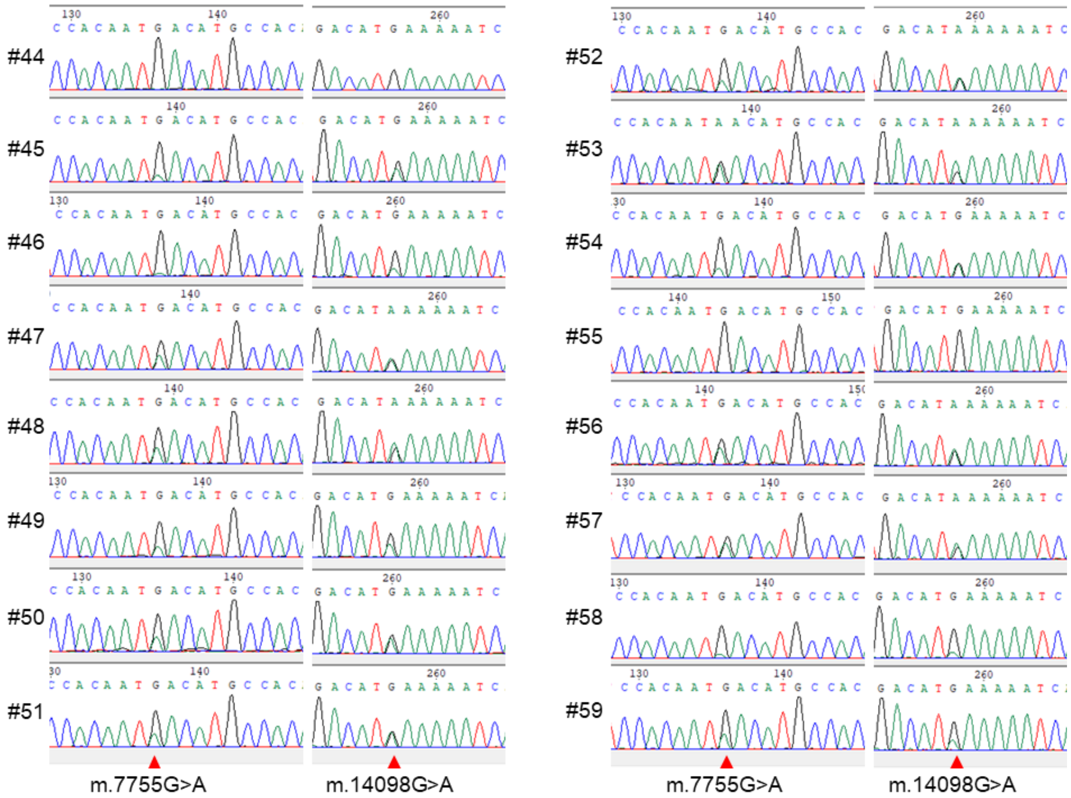

Figure S4. Mutation load in DdCBE-mediated mtDNA two-site mutant F_1 rats.

Sanger sequencing chromatograms of the m.7755G and m.G1498G sites in the toes of sixteen F_1 rats.

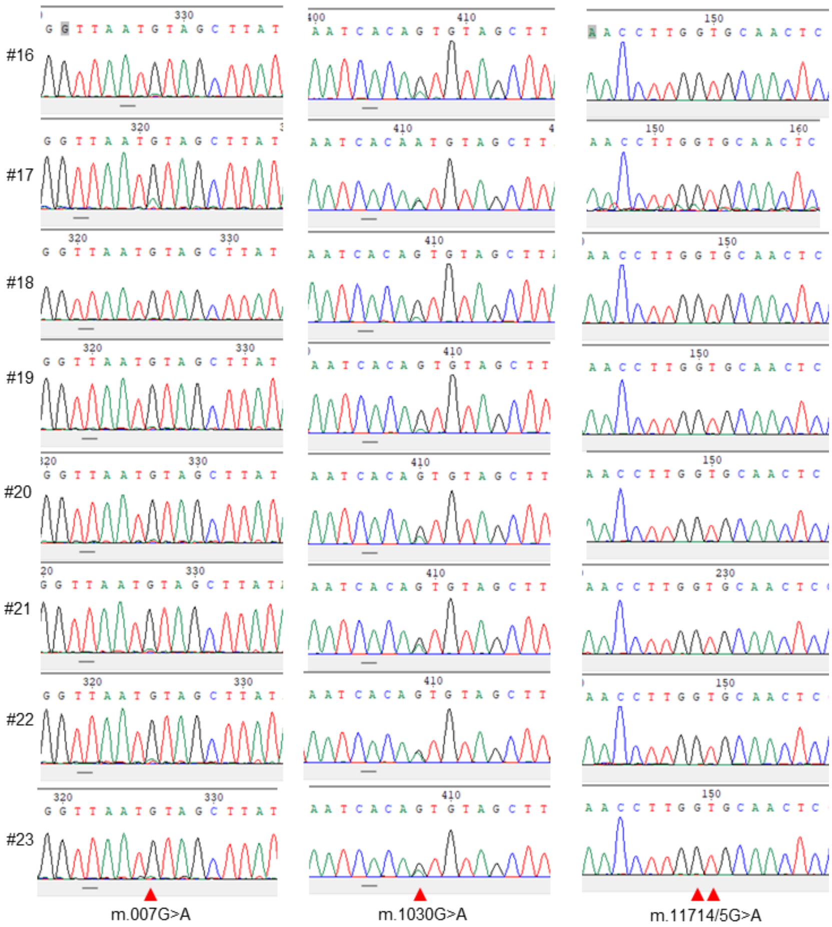

Figure S5. Mutation load in DdCBE-mediated mtDNA three-sites mutant F_1 rats.

Sanger sequencing chromatograms of the m.007G, m.1030G, and m.11714/5G sites in the toes of eight F_1 rats.

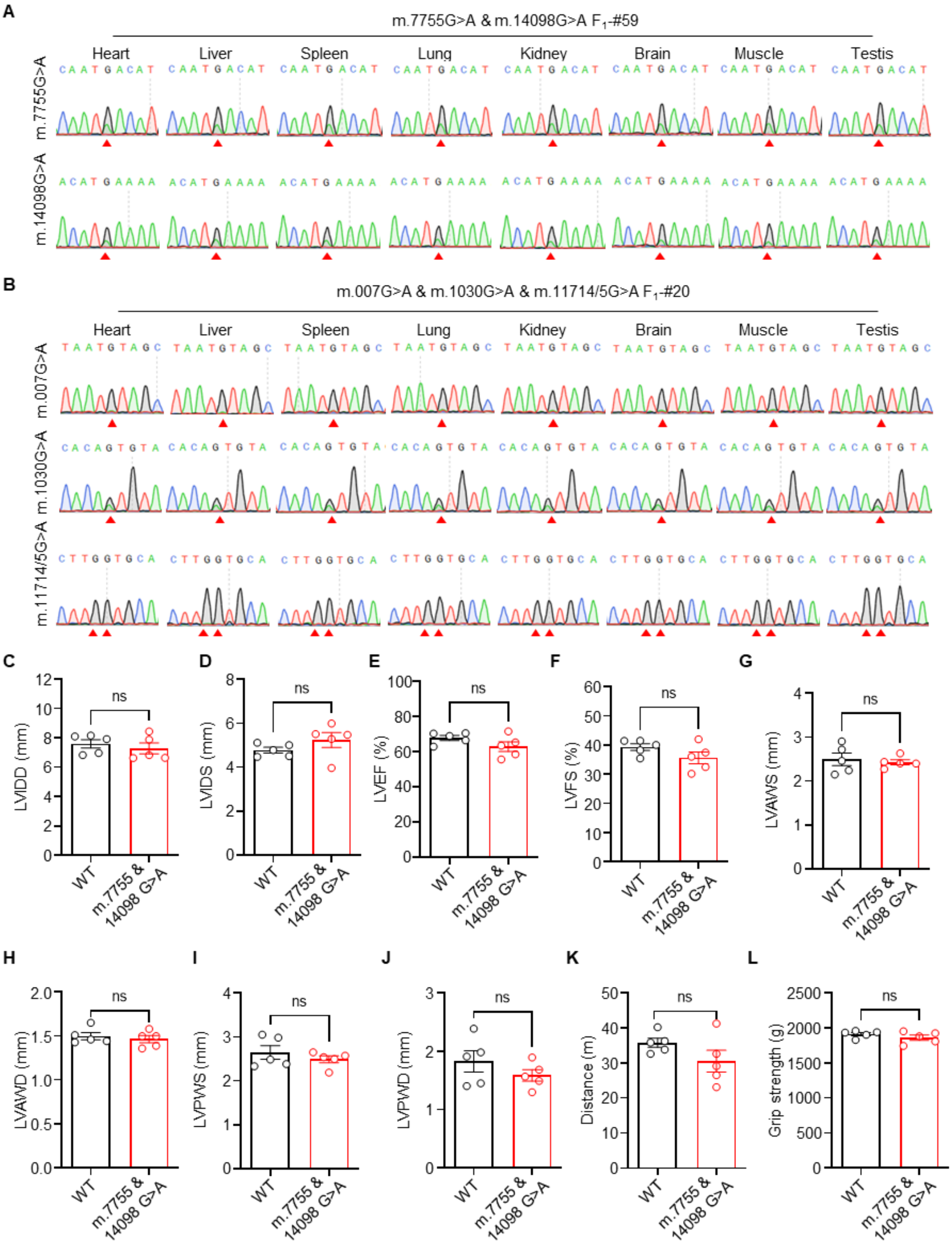

Figure S6. Mutation load and heart function in mtDNA multiple site-edited F_1 rats.

(A) Sanger sequencing chromatograms of the m.7755G, and m.14098G sites in the heart, liver, spleen, lung, kidney, brain muscle, testis of the double mutant F_1-#59 rat.

(B) Sanger sequencing chromatograms of the m.007G, m.1030G, and m.11714/5G sites in the heart, liver, spleen, lung, kidney, brain muscle, testis of the triple mutant F_1-#20 rat.

(C-J) The cardiac structure and function parameters of double mutant F_1 and WT rats. Left ventricular (LV) inner diameter at end of systole (LVIDS) (C) and diastole LVVD (D); LV ejection fraction (LVEF) (E); LV percentage fractional shortening (LVFS) (F); LV anterior wall thickness at end of systole (LVAWS) (G) and diastole (LVAWD) (H); LV posterior wall thickness at end of systole (LVPWS) (I) and

diastole (LVPWD) (J). N=5 for each group; quantitative data were analyzed with the two-tailed unpaired Student's *t*-test and are presented as means ± SD. ns, non-significant.

(K) The distance of WT and double mutant F_1 rats moved in the open field test. N=5 for each group; quantitative data were analyzed with the two-tailed unpaired Student's *t*-test and are presented as means ± SD. ns, non-significant.

(L) The grip strength of WT and double mutant F_1 rats. N=5 for each group; quantitative data were analyzed with the two-tailed unpaired Student's *t*-test and are presented as means ± SD. ns, non-significant.

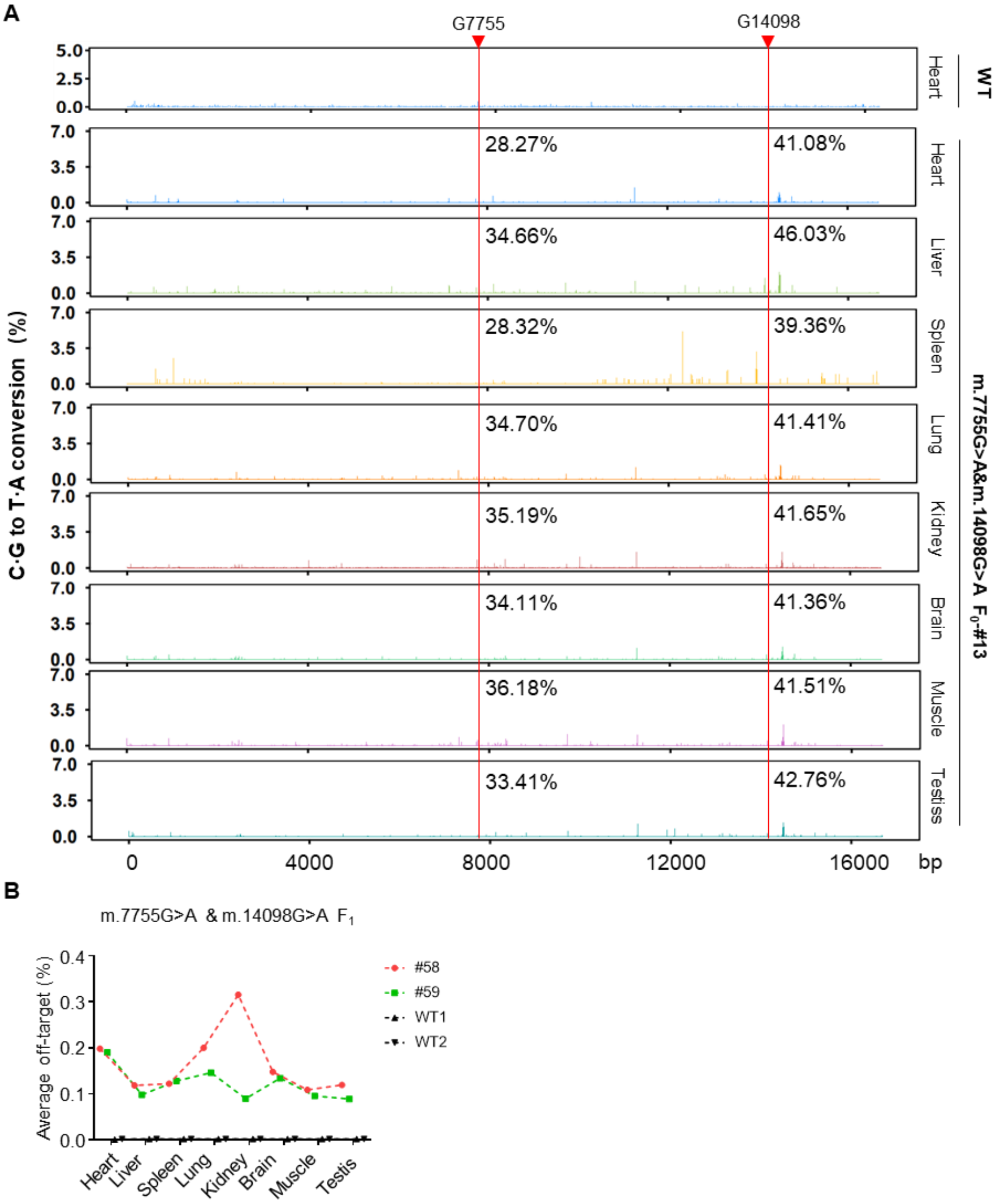

Figure S7. Off-target analysis in the whole mitochondria genome of double-site edited rats.

(A) Off-target effects were analyzed on the whole mitochondrial genome in double mutant F_0-#13 and WT rats. Only sites with over 0.2% editing efficiency in any sample are shown.

(B) Average frequency of mitochondrial genome-wide C• G-to-T •A conversions in double mutant F_1 and WT rats.

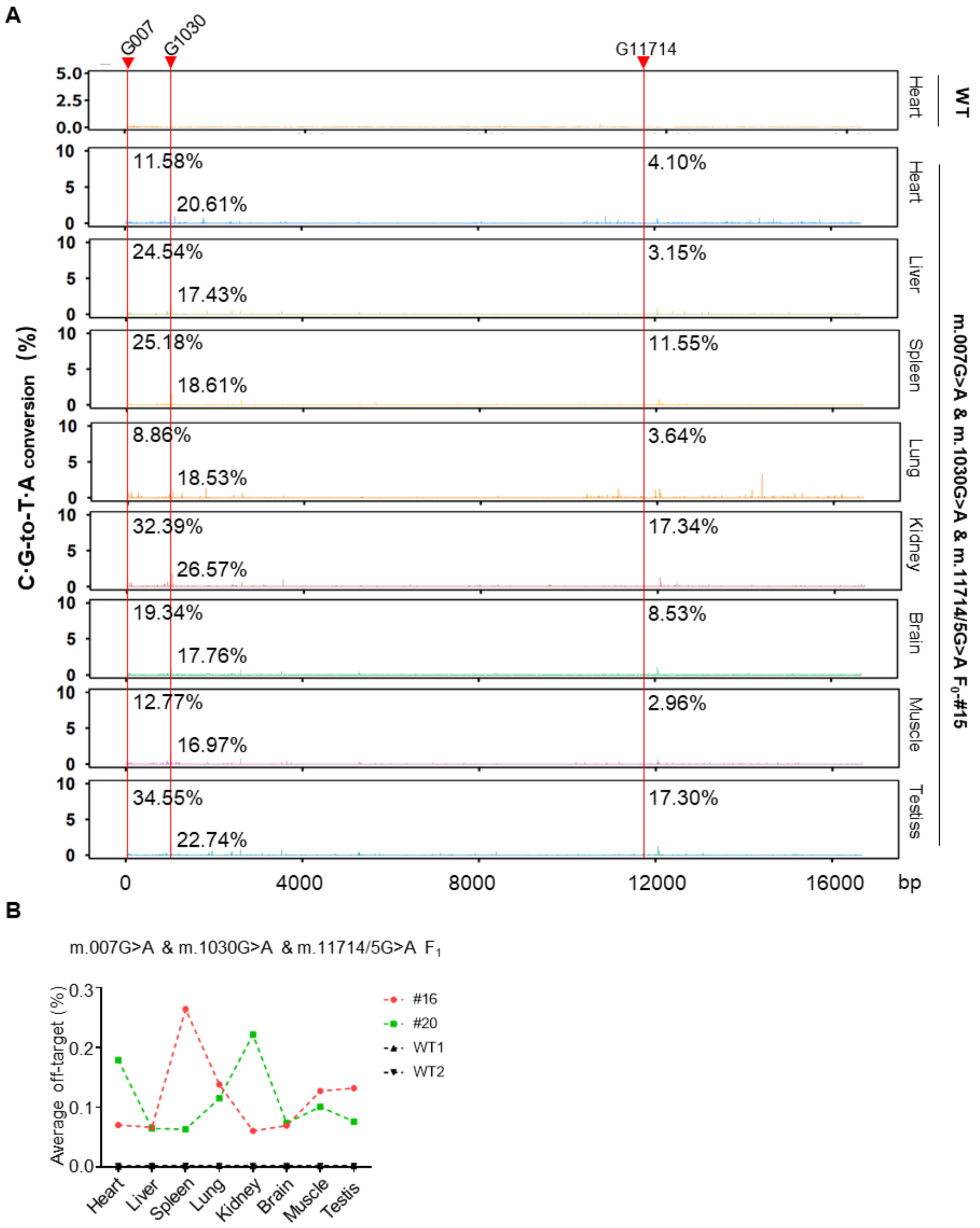

Figure S8. Off-target analysis in the whole mitochondria genome of triple-site edited rats.

(A) Off-target effects were analyzed on the whole mitochondrial genome in triple mutant F_0-#15 and WT rats. Only sites with over 0.2% editing efficiency in any sample are shown.

(B) Average frequency of mitochondria genome-wide C• G-to-T •A conversions in triple mutant F_1 and WT rats.

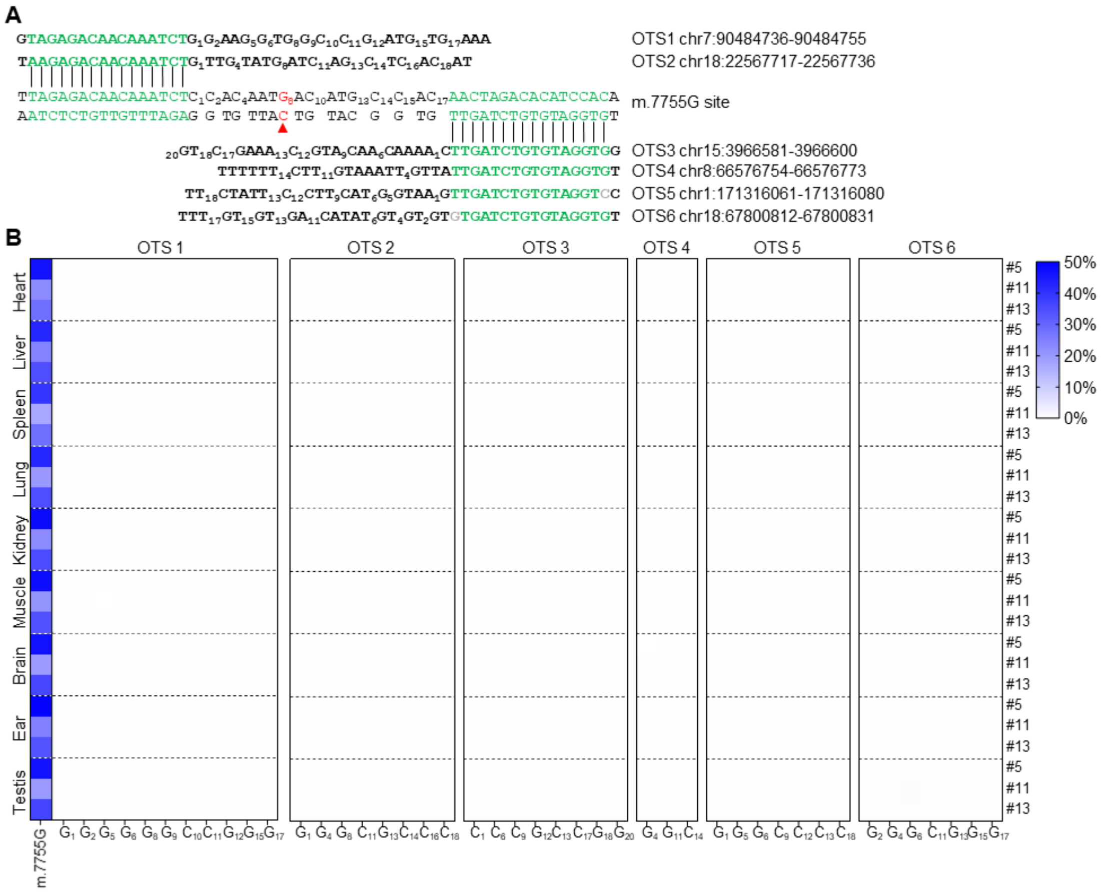

Figure S9. Nuclear genome off-target analysis of m.7755G targeting DdCBE in double mutant rats.

(A) Sequence alignment between rat m.7755G TALE binding sites and potential off-target sites in nuclear genome.

(B) The frequency of C•G-to-T•A conversions at potential nuclear off-target sites in heart, liver, spleen, lung, kidney, muscle, brain, ear, and testis of double mutant F_0 rats.

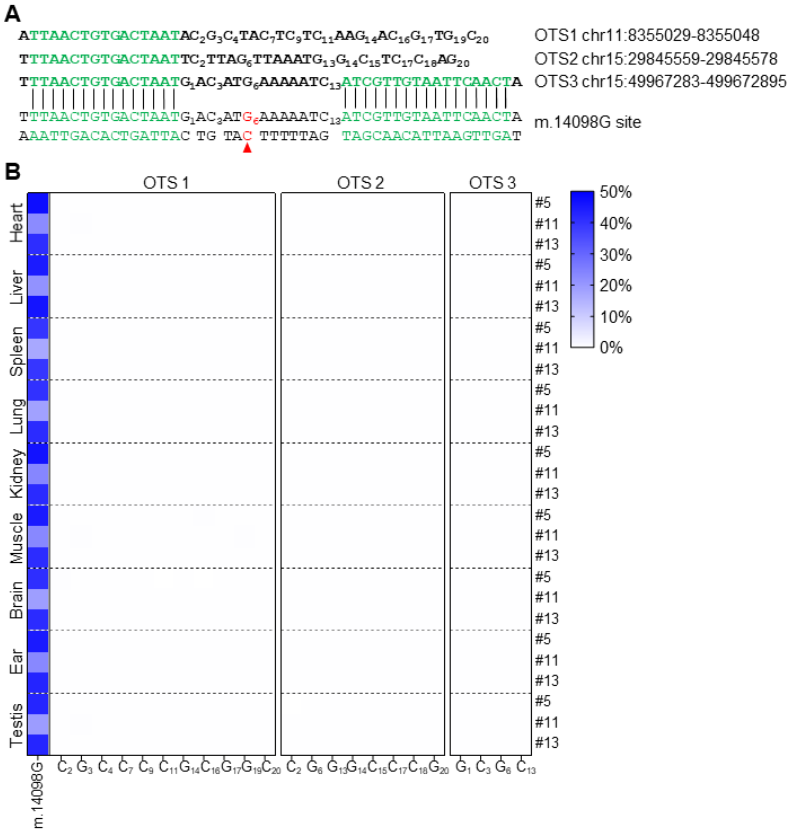

Figure S10. Nuclear genome off-target analysis of m.14098G targeting DdCBE in double mutant rats.

(A) Sequence alignment between rat m.14098G TALE binding sites and potential off-target sites in nuclear genome.

(B) The frequency of C•G-to-T•A conversions at potential nuclear off-target sites in heart, liver, spleen, lung, kidney, muscle, brain, ear, and testis of double mutant F_0 rats.

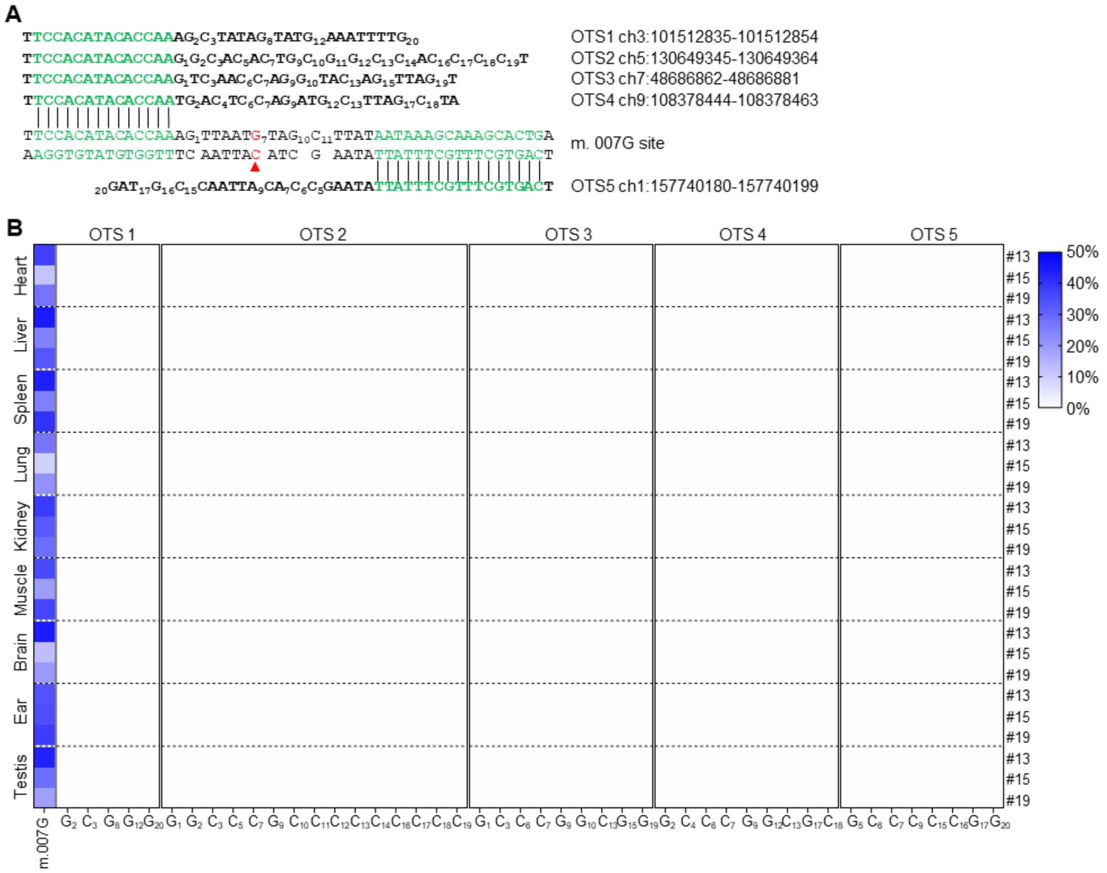

Figure S11. Nuclear genome off-target analysis of m.007G targeting DdCBE in triple mutant rats.

(A) Sequence alignment between rat m.007G TALE binding sites and potential off-target sites in nuclear genome.

(B) The frequency of C•G-to-T•A conversions at potential nuclear off-target sites in heart, liver, spleen, lung, kidney, muscle, brain, ear, and testis of triple mutant F_0 rats.

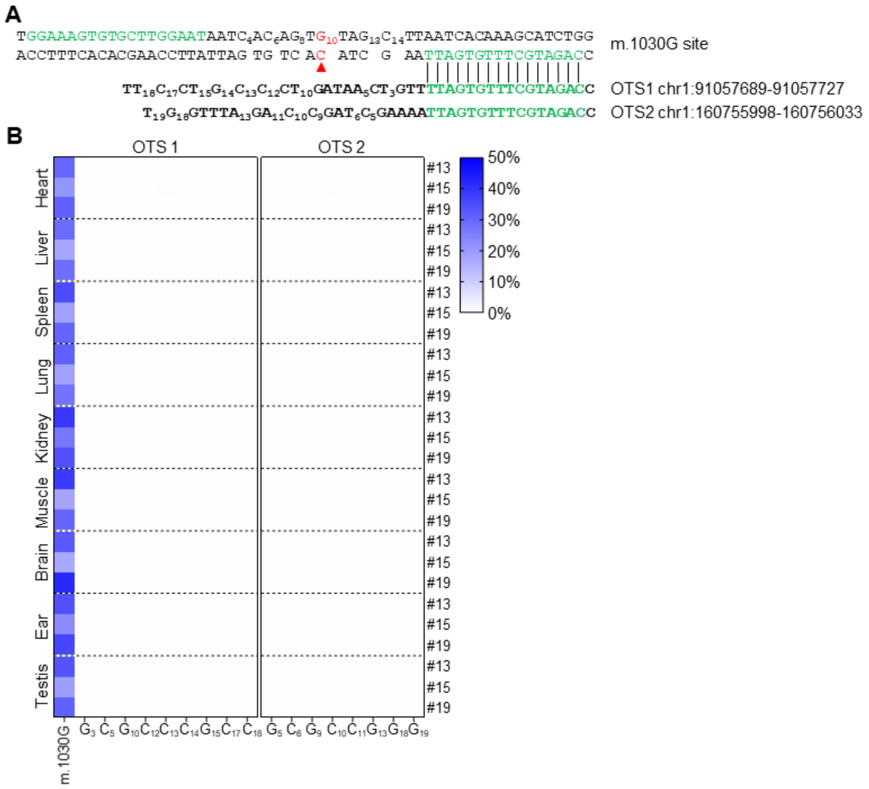

Figure S12. Nuclear genome off-target analysis of m.1030G targeting DdCBE in triple mutant rats.

(A) Sequence alignment between rat m.1030G TALE binding sites and potential off-target sites in nuclear genome.

(B) The frequency of C•G-to-T•A conversions at potential nuclear off-target sites in heart, liver, spleen, lung, kidney, muscle, brain, ear, and testis of triple mutant F_0 rats.

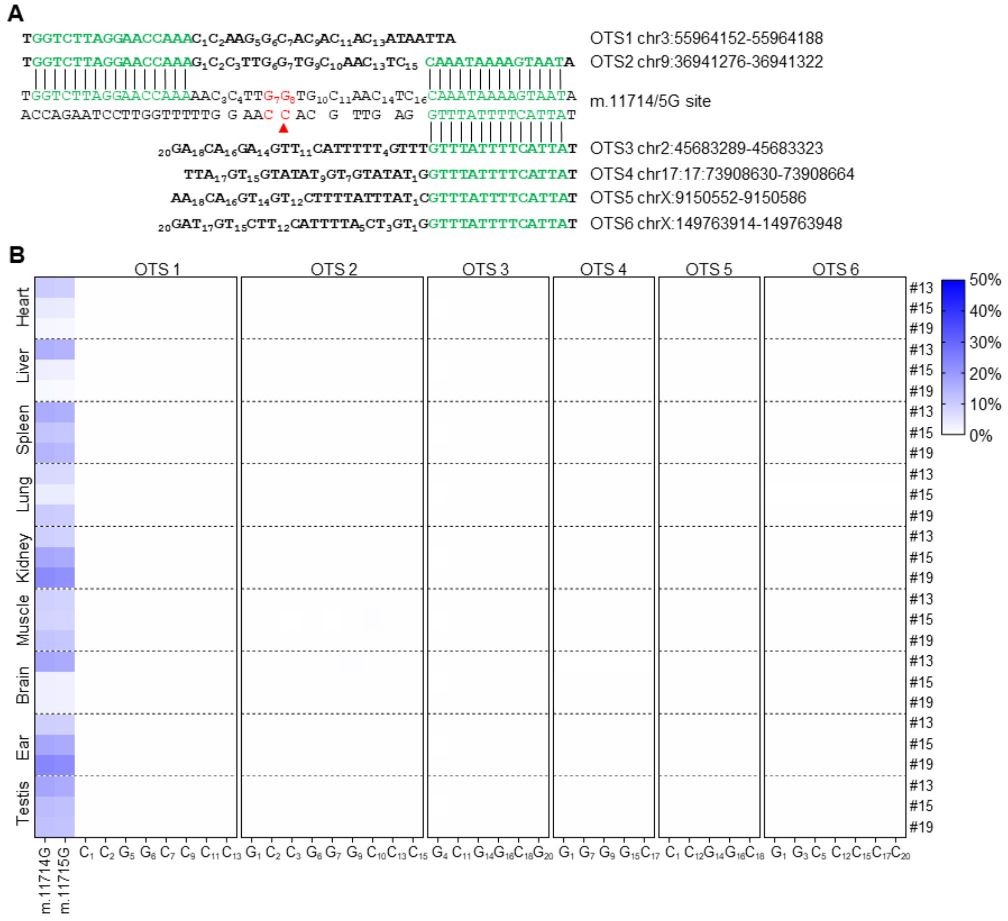

Figure S13. Nuclear genome off-target analysis of m.11714/5G targeting DdCBE in triple mutant rats.

(A) Sequence alignment between rat m.11714/5G TALE binding sites and potential off-target sites in nuclear genome.

(B) The frequency of C•G-to-T•A conversions at potential nuclear off-target sites in heart, liver, spleen, lung, kidney, muscle, brain, ear, and testis of triple mutant F_0 rats.

Tables

Table S1. Germline transmission summary of F_0 mutant rats.

Target site	Founder rat	No. of F_1	No. of edited F_1 (rate)	Mutation load
m.007G &<br>m.1030G &<br>m.11714/5G	#22	8	8 (100%)	9.21%-19.32%<br>17.78%-54.05%<br>9.8%
m.7755G &<br>m.14098G	#10	16	14 (87.50%)	6.85%-47.11%<br>18.52-64.29%

Table S2. Primers used in this study.

	Primers	Sequence (5' - 3')
Primers for genotyping	Rat-G7755-Fwd	TGCTCTGAAATTTGCGGCTC
	Rat-G7755-Rev	AGTCGGTTGCTGATTAGGCG
	Rat-G14098-Fwd	TCAAGTCTCCGGGTACTCCT
	Rat-G14098-Rev	AAATATTGAGGCGCCGTTGG
	Rat-G007-Fwd	AGGCATCTGGTTCTTACTTCAGG
	Rat-G007-Rev	TTATTAAGGTTTAGGGCTAAGCATAGTGG
	Rat-G1030-Fwd	GCGGTACTTTATATCCATCTAGAGG
	Rat-G1030-Rev	GTTTTTAGGTAGCTCGTTTGGTTTC
	Rat-G11714/5-Fwd	TGAATCTAACAACAGGAAATCAAATTC
	Rat-G11714/5-Rev	GATATAATTCCTACTCCTTCTCATCCAAT
Barcoded Primers for on-target Deep-sequence	i5-rat-G7755-Fwd1	ACACTCTTTCCCTACACGACGCTCTTCCGATCT CAAAACTCATTGCGAAGCTTAGAGCG
	i5-rat-G7755-Fwd2	ACACTCTTTCCCTACACGACGCTCTTCCGATCTT CAAACTCATTGCGAAGCTTAGAGCG
	i5-rat-G7755-Fwd3	ACACTCTTTCCCTACACGACGCTCTTCCGATCT ACCAACTCATTGCGAAGCTTAGAGCG
	i7-rat-G7755-Rev	GTGACTGGAGTTCAGACGTGTGCTCTTCCGATC TTGTGGCTATAGTTTTGGGGGAGG
	i5-rat-G14098-Fwd1	ACACTCTTTCCCTACACGACGCTCTTCCGATCT CAAAGCCCACCATAAATAGGTGAAGG
	i5-rat-G14098-Fwd2	ACACTCTTTCCCTACACGACGCTCTTCCGATCTT CAAGCCCACCATAAATAGGTGAAGG
	i5-rat-G14098-Fwd3	ACACTCTTTCCCTACACGACGCTCTTCCGATCT ACCAGCCCACCATAAATAGGTGAAGG
	i7-rat-G14098-Rev	GTGACTGGAGTTCAGACGTGTGCTCTTCCGATC TTAGGGGGTGAGATTTTCGGAT
	i5-Rat007 Fwd1	ACACTCTTTCCCTACACGACGCTCTTCCGATCT GAGGCATTGGTAAAATTTCCCGACA
	i5-Rat007 Fwd2	ACACTCTTTCCCTACACGACGCTCTTCCGATCTT AAGCATTGGTAAAATTTCCCGACA
	i5-Rat007 Fwd3	ACACTCTTTCCCTACACGACGCTCTTCCGATCTT CAGCATTGGTAAAATTTCCCGACA
	i7-Rat007 Rev1	GTGACTGGAGTTCAGACGTGTGCTCTTCCGATC TACCCACCGGTTTATGGATGTTTGC
	i7-Rat007 Rev2	GTGACTGGAGTTCAGACGTGTGCTCTTCCGATC TCAGCACCGGTTTATGGATGTTTGC
	i7-Rat007 Rev3	GTGACTGGAGTTCAGACGTGTGCTCTTCCGATC TCCTCACCGGTTTATGGATGTTTGC
	i5-Rat1030 Fwd1	ACACTCTTTCCCTACACGACGCTCTTCCGATCT GAGCAATGAAGTACGCACACACCG
	i5-Rat1030 Fwd2	ACACTCTTTCCCTACACGACGCTCTTCCGATCTT AACAATGAAGTACGCACACACCG
	i5-Rat1030 Fwd3	ACACTCTTTCCCTACACGACGCTCTTCCGATCTT CACAATGAAGTACGCACACACCG
	i7-Rat1030 Rev1	GTGACTGGAGTTCAGACGTGTGCTCTTCCGATC TACCTGTTGGTTGGTTGTAGGGCT
	i7-Rat1030 Rev2	GTGACTGGAGTTCAGACGTGTGCTCTTCCGATC TCAGTGTTGGTTGGTTGTAGGGCT
	i7-Rat1030 Rev3	GTGACTGGAGTTCAGACGTGTGCTCTTCCGATC TCCTTGTTGGTTGGTTGTAGGGCT
	i5-Rat11714/5 Fwd1	ACACTCTTTCCCTACACGACGCTCTTCCGATCT GAGGAACTGCTAATTCATGCACCCAT

	i5-Rat11714/5 Fwd2	ACACTCTTTCCCTACACGACGCTCTTCCGATCTT AAGAACTGCTAATTCATGCACCCAT
	i5-Rat11714/5 Fwd3	ACACTCTTTCCCTACACGACGCTCTTCCGATCTT CAGAACTGCTAATTCATGCACCCAT
	i7-Rat11714/5 Rev1	GTGACTGGAGTTCAGACGTGTGCTCTTCCGATC TACCGGTAAGAGGCTAAGGAGGAATG
	i7-Rat11714/5 Rev2	GTGACTGGAGTTCAGACGTGTGCTCTTCCGATC TCAGGGTAAGAGGCTAAGGAGGAATG
	i7-Rat11714/5 Rev3	GTGACTGGAGTTCAGACGTGTGCTCTTCCGATC TCCTGGTAAGAGGCTAAGGAGGAATG
Primers for long-range PCR	rat-mt 2Mix Fwd	TGGCTTACAAGACGCCACAT
	rat-mt 2Mix Rev	GTGGGCGGGTTGTTGATTTC
	rat-mt 3Mix Fwd	TCGCTCCCACTTAATATCTACTCT
	rat-mt 3Mix Rev	TTAGTCACTGGGCAGGCAATG

mitoTALE amino acid sequences

Amino acids of DdCBEs using for rat m.7755G, m.14098G, m.G007G, m.G1606G and m.G11714/5G sites editing are listed as separated protein sequence file:

red for MTS, italics for linker, yellow for flag tag, green for N&C-terminal domain, gold for RVDs, purple for split $DddA_{tox}$ halves, cyan for UGI.

Rat m. 7755G to A TALE-L15-G1397N.prot
Rat m. 7755G to A TALE-R16-G1397C.prot
Rat m. 14098G to A TALE-L15-G1397C.prot
Rat m. 14098G to A TALE-R17-G1397N.prot
Rat m. 007G to A TALE-L14-G1333C.prot
Rat m. 007G to A TALE-R17-G1333N.prot
Rat m. 1030G to A TALE-L18-G1333C.prot
Rat m. 1030G to A TALE-R16-G1333N.prot
Rat m. 11714/5G to A TALE-L16-G1333C.prot
Rat m. 11714/5G to A TALE-R14-G1333N.prot
